# Opposing and Complementary Topographic Connectivity Gradients Revealed by Quantitative Analysis of Canonical and Noncanonical Hippocampal CA1 Inputs

**DOI:** 10.1523/ENEURO.0322-17.2018

**Published:** 2018-01-30

**Authors:** Yanjun Sun, Douglas A. Nitz, Todd C. Holmes, Xiangmin Xu

**Affiliations:** 1 Department of Anatomy and Neurobiology, School of Medicine, University of California, Irvine, CA 92697-1275; 2 Department of Cognitive Science, University of California, San Diego, La Jolla, CA 92093; 3 Department of Physiology and Biophysics, School of Medicine, University of California, Irvine, CA 92697-4560; 4 Department of Biomedical Engineering, University of California, Irvine, CA 92697-2715; 5 Department of Microbiology and Molecular Genetics, School of Medicine, University of California, Irvine, CA 92697-4025; 6 Department of Computer Science, University of California, Irvine, CA 92697-3435

**Keywords:** Circuit connections, hippocampus, imaging, quantitative, viral tracing

## Abstract

Physiological studies suggest spatial representation gradients along the CA1 proximodistal axis. To determine the underlying anatomical basis, we quantitatively mapped canonical and noncanonical inputs to excitatory neurons in dorsal hippocampal CA1 along the proximal-distal axis in mice of both sexes using monosynaptic rabies tracing. Our quantitative analyses show comparable strength of subiculum complex and entorhinal cortex (EC) inputs to CA1, significant inputs from presubiculum and parasubiculum to CA1, and a threefold stronger input to proximal versus distal CA1 from CA3. Noncanonical subicular complex inputs exhibit opposing topographic connectivity gradients whereby the subiculum-CA1 input strength systematically increases but the presubiculum-CA1 input strength decreases along the proximal-distal axis. The subiculum input strength cotracks that of the lateral EC, known to be less spatially selective than the medial EC. The functional significance of this organization is verified physiologically for subiculum-to-CA1 inputs. These results reveal a novel anatomical framework by which to determine the circuit bases for CA1 representations.

## Significance Statement

In the present work, we applied an unbiased Cre-dependent rabies tracing system for mapping and comparing canonical CA3/EC and noncanonical subiculum (SUB) complex inputs to excitatory pyramidal neurons of CA1 in the intact brain. We reveal previously unrecognized features of both canonical and noncanonical hippocampal circuitry. The observed contrast in afferent sources for distal versus proximal CA1 pyramidal neurons indicates that the transverse-axis division of the canonical trisynaptic pathway must be considered as input biases of MEC and CA3 to proximal CA1 versus input biases of SUB and LEC to distal CA1. Our novel results outline a new framework that will be critical to understanding how distinct transverse-axis hippocampus/EC circuits contribute to learning, memory, and spatial cognition.

## Introduction

The hippocampal formation (HPC) and associated entorhinal cortices (EC) are critical to learning and memory processes (O’Keefe, 1976; Ergorul and Eichenbaum, 2004; Hafting et al., 2005; Tse et al., 2007; Moser et al., 2008). Connectivity patterns among the HPC subregions CA3, CA2, CA1, subiculum (SUB), dentate gyrus (DG) and the medial and lateral entorhinal cortices (MEC and LEC, respectively) specify distinct circuits that guide research into HPC function and neurophysiology (Henriksen et al., 2010; Burke et al., 2011; Nakamura et al., 2013; Igarashi et al., 2014; Lee et al., 2015; Lu et al., 2015; Sun et al., 2017a). Historically, such work has been dominated by consideration of stages along the trisynaptic circuit, now commonly referred as the “canonical” hippocampal circuit (Amaral and Witter, 1989; Ishizuka et al., 1990; Witter, 2007; Xu et al., 2016).

Although HPC and EC neurons throughout the canonical circuit have action potential firing correlates to many variables, the correlations of firing to position and/or orientation within an environment are particularly strong and have proven to be a powerful way to assess learning, memory, and spatial cognition functions of HPC and MEC/LEC. Combined with assessments of the strength of connectivity between structures, the differences in neural dynamics have led to important concepts regarding each region’s functional role in spatial and temporal pattern separation, pattern completion, memory recall, and navigation. More recently, the functional division of the trisynaptic, or canonical, HPC circuit along the transverse axis of HPC and EC has been revisited according to the division of EC into lateral (LEC) and medial (MEC) components and division of the CA3, CA1, and SUB cell body layers into distal and proximal components (Witter et al., 1990; Henriksen et al., 2010; Burke et al., 2011; Kim et al., 2012; Nakamura et al., 2013; Igarashi et al., 2014; Lee et al., 2015; Lu et al., 2015; Sun et al., 2017a). Multiple anatomical studies evidence splitting of the canonical circuit into two pathways. The first pathway leads from layer II LEC to DG (outer molecular layer) to CA3 to distal CA1, and, finally, to proximal SUB. Here, distal CA1 and proximal SUB project back to layer V LEC. The second pathway leads from layer II MEC to DG (middle molecular layer) to CA3 to proximal CA1, and finally, to distal SUB. Proximal CA1 and distal SUB form return projections into layer V MEC. Within these circuits, it is logical to include the direct projections from layer III MEC and LEC into, respectively, proximal and distal CA1. Furthermore, advances in understanding the functional significance of transverse axis divisions of the canonical HPC circuit indicate that characterization of the circuit itself may be incomplete. Earlier and more recent studies have identified a noncanonical “reverse” pathway from SUB to CA1 in different species (Berger et al., 1980; Seress et al., 2002; Jackson et al., 2014; Sun et al., 2014; Xu et al., 2016).

In the present work, we applied a Cre-dependent rabies tracing system that we developed earlier (Sun et al., 2014) for precise quantification of connection strengths between HPC and EC subregions. We mapped and compared canonical CA3/EC and noncanonical subiculum complex inputs to excitatory pyramidal neurons of CA1. We reveal previously unrecognized features of both canonical and noncanonical HPC circuitry. These include the presence of a significant input from presubiculum to proximal CA1, a larger than threefold difference in the strength of CA3 to proximal CA1 projections compared with CA3 to distal CA1 projections, an approximate 35% overall difference in the strength of MEC versus LEC direct inputs to CA1, and approximate equality in the strength of SUB inputs to distal CA1 compared to combined MEC/LEC inputs to distal CA1. The impact of noncanonical SUB to CA1 projections is evidenced in *in vitro* CA1 recordings in which caged glutamate is used to stimulate SUB. Together, the findings outline a new framework likely critical to understanding how distinct transverse-axis HPC/EC circuits contribute to learning, memory, and spatial cognition.

## Materials and Methods

### Animals

All experiments were conducted according to National Institutes of Health guidelines for animal care and use and were approved by the Institutional Animal Care and Use Committee of the University of California, Irvine. Although the genetically modified rabies viruses used for the proposed experiments are deletion-mutant rabies and are based on a vaccine strain (SAD-B19), they still pose a limited potential health risk with the helper virus. All personnel working with the rabies are therefore vaccinated and experiments are conducted under biosafety level (BSL) 2 conditions with a protocol approved by the institutional biosafety committee.

To achieve Cre-directed, cell type–specific expression of TVA receptors in hippocampal CA1, we used a LSL-R26^Tva-lacZ^ mouse line conditionally expressing TVA receptor (avian retroviral receptor, tumor virus A) in a Cre-recombinase-dependent manner (Seidler et al., 2008); the LSL-R26^Tva-lacZ^ mouse line was cross-bred with Camk2a-Cre (T29) mouse line (Tsien et al., 1996) to target cortical excitatory neurons. We termed the double transgenic mice as Camk2a-Cre; TVA, in which Cre-expressing cells also express TVA to restrict initial infection of EnvA-SADΔG rabies virus. Mice of ∼12 wk old (either sex) were used for experiments and had free access to food and water in their home cages before and after surgeries.

### Viral injections for neural circuit tracing

To perform stereotaxic viral injections into the brain, mice were anesthetized under 1.5% isoﬂurane for 10 min with a 0.8 L/min oxygen flow rate using an isoﬂurane table top unit (HME109, Highland Medical Equipment). Mice were then placed in a rodent stereotax (Leica Angle Two for mouse) with continuous 1% isoflurane anesthesia with the head secured. A small incision was made in the head, the skin was reflected, and the skull was exposed to show the landmarks of bregma and lambda, and desired injection sites. A three-axis micromanipulator guided by a digital atlas was used to determine coordinates for the bregma and lambda. The following injection coordinates targeting different brain regions were used (all values given relative to the bregma), intermediate CA1: anteroposterior (AP) −2.06 mm, lateromedial (ML) –1.40 mm; dorsoventral (DV) −1.35 mm; proximal CA1: AP –2.06 mm, ML –1.94 mm, DV –1.51 mm; distal CA1: AP –2.06 mm, ML –0.85 mm, DV –1.38 mm. A small drill hole was made in the skull over the injection site, exposing the pia surface. A pulled-glass pipette (tip diameter, ≈30 μm) was loaded with virus and then lowered into the brain with the appropriate coordinates. A Picospritzer (General Valve) was used to pulse virus into the brain. A total of 0.1 µl of the helper virus (AAV8-EF1a-DIO-HB, ∼2 × 10^11^ genome units per ml; Addgene, Plasmid 37452) was injected into the brain of Camk2a-Cre; TVA mouse at a rate of 20–30 nl/min, with 10-ms pulse duration. For some of the cases, the AAV helper virus was delivered into the brain through iontophoresis with a positive 3-µA current at 7-s on and 7-s off cycles for 5–8 min. To prevent backﬂow of virus, the pipette remained in the brain for 5 min after completion of the injection. Once the injection pipette was withdrawn, the mouse was removed from the stereotaxis, and the incision was closed with either wound clips or tissue adhesive (3M Vetbond). Mice were taken back and recovered in their home cages. After 3 wk of the AAV injection, which allowed for the infected neurons to express high contents of RGs and GFP, a pseudotyped, RG-deleted rabies virus (EnvA-SADΔG-mCherry rabies, 0.4 µl, ∼3 × 10^8^ infectious units per ml; or EnvA-SADΔG-mCherry rabies, 0.1 µl, ∼2 × 10^9^ infectious units per ml) was injected into the same location as the AAV injections via the Picospritzer. The rabies virus was allowed to replicate and retrogradely spread from targeted Cre^+^ cell types to directly connected presynaptic cells for 9–10 d before the animals were perfused for tissue processing. Because it has been estimated that rabies virus requires only 24 h to cross a synapse (Ugolini, 2008), the rabies infection time would be sufficient for crossing sparse synaptic contacts, which is confirmed by our results.

Note that our use of the EnvA-TVA system and EnvA-pseudotyped SADΔG rabies largely overcomes the tropism issue related to wild-type enveloped SADΔG rabies in neural circuit tracing studies reported by Albisetti et al. (2017). Although Beier et al. (2017) show that the EnvA-SADΔG rabies-based tracing allows for unbiased mapping of input populations, they used a shorter incubation time of 5 d to better detect input plasticity of the ventral tegmental area (Beier et al., 2017). To compensate for the potential time differences of transynaptic propagation across strong versus weak synapses, we have used rabies incubation times of 9–10 d to allow for ample times of monosynaptic crossings and genetic label expression in presynaptic input neurons to reduce biased mapping (Beier et al., 2017). Furthermore, although input activity can enhance rabies propagation (Beier et al., 2017), our experimental conditions allowed for robust mapping of inputs from different neuron types with different *in vivo* firing rates.

### Histology and immunohistochemistry

The mice were transcardially perfused with 5 ml PBS, followed by 25 ml PBS containing 4% paraformaldehyde. The brains were removed and left in 4% paraformaldehyde overnight, then transferred into 30% sucrose in PBS in the next day. The brains were sectioned coronally at 30-µm thickness on a freezing microtome (Leica SM2010R). To better examine both the laminar structure of the entorhinal cortex and the CA1 injection site, for some of the cases, we used a combined coronal/horizontal sectioning technique described in Steward (1976). Basically, the brains were divided with a coronal cut at approximately the posterior border of the dorsal psalterium, and the rostral portion of the brain was sectioned in the coronal plane, whereas the caudal region was sectioned in the horizontal plane. Every one of three sections was mounted for examination and quantification of starter cells and their presynaptic cells in different brain structures. Sections were counterstained with 10 µM DAPI for better visualization of cortical and subcortical structures.

As the GFP expression of AAV and mCherry expression of rabies are strong in labeled cells, we did not perform immunostaining against either GFP or mCherry. Selected presubiculum sections were immunostained with parvalbumin (PV) and calbindin-D28K (CB) antibodies for distinguishing the entorhinal cortex from pre/parasubiculum (Fujise et al., 1995; Fujimaru and Kosaka, 1996). Conventional immunochemistry was performed as described previously (Xu et al., 2010b). CB immunostaining was performed with a rabbit anti-CB primary antibody (Swant; RRID:AB_10000340; 1:1000) followed with an AF647-conjugated donkey anti-rabbit secondary antibody (Jackson ImmunoResearch, 1:200). For PV staining, a goat anti-PV primary antibody (Swant; RRID:AB_2650496; 1:1000) was followed with an AF488-conjugated donkey anti-goat secondary antibody (Jackson ImmunoResearch, 1:200). For reelin staining, a mouse anti-reelin monoclonal primary antibody (MBL; RRID:AB_843523; 1:200) was followed with an AF488-conjugated donkey anti-mouse secondary antibody (Jackson ImmunoResearch, 1:200). Sections were counterstained with 10 μm DAPI, then mounted and coverslipped with a Vectashield antifade mounting medium (Vector Laboratories).

### Image data acquisition and analysis

Brain section images were acquired by using automated slide scanning and analysis software (MetaMorph) in a high-capacity computer coupled with a fluorescent BX61 Olympus microscope and a high-sensitive Hamamatsu CCD camera. Under a 10× objective, we were able to obtain images of sufficient resolution for all subsequent computer-based analyses. Image stitching, overlaying, cell counting, and further imaging analysis were completed by using MetaMorph imaging and analysis tools. In addition, we imaged labeled cells in selected sections with a confocal microscope (LSM 700/780, Carl Zeiss Microscopy) coupled with *z*-stack and tile scanning features under a 20× objective lens. Image stitching, overlaying, maximum projections, and export were performed by using the ZEN software analysis tools.

Quantitative examinations across the series of sections were conducted for complete and unbiased analyses of rabies-mediated, direct synaptic connections to targeted Cre-defined cell types by using either MetaMorph or Adobe Photoshop software (CS4 extended version, Adobe Systems). For mapping rabies-labeled presynaptic neurons (expressing mCherry only), digital images of brain sections were examined to identify and mark the locations of mCherry-expressing cell bodies. These labeled cells were assigned to specific anatomic structures for regional input quantification.

### Fast voltage-sensitive dye imaging

C57BL/6J mice were deeply anesthetized with pentobarbital sodium (>100 mg/kg, i.p.) and rapidly decapitated, and their brains were removed. Following the protocol in Kopanitsa et al. (2006), hippocampal slices 400 µm thick were cut at an angle of 20–30° in the horizontal plane to conserve the intrahippocampal axonal projections in well-oxygenated (95% O_2_-5% CO_2_), ice-cold sucrose-containing cutting solutions (in mm: 85 NaCl, 75 sucrose, 2.5 KCl, 25 glucose, 1.25 NaH_2_PO_4_, 4 MgCl_2_, 0.5 CaCl_2_, and 24 NaHCO_3_). Two slices prepared at appropriate levels of the dorsal-ventral axis of hippocampus from each hemisphere were visually confirmed to have preserved CA1-SUB structures, which were then used for experiments. For voltage-sensitive dye (VSD) imaging experiments, slices were first incubated in the cutting solution for 30 min at 32°C, and then transferred for dye staining at room temperature (22°C) for 1 h in oxygenated ACSF (in mm: 126 NaCl, 2.5 KCl, 26 NaHCO_3_, 2 CaCl_2_, 2 MgCl_2_, 1.25 NaH_2_PO_4_, and 10 glucose) containing 0.12 mg/ml of the absorption voltage-sensitive dye, NK3630 (Kankoh-Shikiso Kenkyusho), then maintained in the regular ACSF before use. Throughout the cutting, incubation, and recording processes, the solutions were continuously supplied with 95% O_2_-5% CO_2_. We used standard open recording chambers which maintained slice health and viability well, as evidenced by the measurement of neural activities for periods lasting >6 h.

Our overall system of electrophysiological recordings, photostimulation, and imaging was described previously (Xu et al., 2010a). The solution was fed into the slice recording chamber through a pressure-driven flow system with pressurized 95% O_2_-5% CO_2_. The perfusion flow rate was ∼2 mL/min. Stock solution of MNI-caged-l-glutamate (Tocris Bioscience) was added to 20 ml ACSF for a final concentration of 0.2 mm caged glutamate. Caged glutamate was present in the bath solution and turned active only through focal UV photolysis. The slice image was acquired by a high-resolution digital CCD camera, which in turn was used for guiding and registering photostimulation sites. A laser unit (DPSS Lasers) was used to generate a 355-nm UV laser for glutamate uncaging. Short pulses of laser flashes (1 ms, 20 mW) were controlled using an electro-optical modulator and a mechanical shutter. The laser beam formed uncaging spots, each approximating a Gaussian profile with a width of ∼100 µm laterally at the focal plane.

During VSD imaging experiments, 705-nm light trans-illuminated brain slices and voltage-dependent changes in the light absorbance of the dye were captured by the MiCAM02 fast imaging system (SciMedia USA). The illumination was supplied by optically filtering white light produced by an Olympus tungsten-halogen lamp up to 100 W. Optical recording of VSD signals was performed under a 4× objective with a sampling rate of 4.4 ms per frame [frame resolution 88 pixels (width × 60 pixels (height)]. The field of view covered up to an area of 1.28 × 1.07 mm^2^ in the hippocampal formation. VSD imaging of evoked activity was triggered and synchronized with each laser photostimulation at specified cortical sites. For each trial, the VSD imaging duration was 2000 frames including 500 baseline frames, with an intertrial interval of 12 s. Each session lasts for 9 × 12 s (108 s total).

VSD signals were originally measured by the percentage change in pixel light intensity [Δ*I*/*I*%; the % change in the intensity (Δ*I*) at each pixel relative to the initial intensity (*I*)]. In addition, the mean and SD of the baseline activity of each pixel across the 50 frames preceding photostimulation was calculated, and VSD signal amplitudes were then expressed as SD multiples above the mean baseline signal for display and quantification. The activated pixel was empirically defined as the pixel with the amplitude ≥1 SD above the baseline mean of the corresponding pixel’s amplitude (equivalent to the detectable signal level in the original VSD maps of Δ*I*/*I*%). VSD images were smoothed by convolution with a Gaussian spatial filter [kernel size, 5 pixels; SD (σ), 1 pixel], and a Gaussian temporal filter (kernel size, 3 frames; σ, 1 frame). In the present study, single-trial VSD signals were of sufficiently high amplitudes and could be discerned from background noise; no averaging over multiple trials was used for data presentation unless specified. Images were displayed and analyzed using custom-made Matlab programs. To quantify VSD response strength of photostimulation-evoked neural activities, the average number of activated pixels and average response amplitude within the defined window of analysis were measured for each trial.

### Statistical analysis

Data are presented as mean ± SE. For statistical comparisons between groups, the data were checked for normality distribution and equal variance. When the criteria were met, a *t* test was performed to compare two groups; when the criteria were not met, a Mann–Whitney *U* test was used. For statistical comparisons across more than two groups, one-way ANOVA with a Tukey *post hoc* test was used for group comparisons. In all experiments, the level of statistical significance was defined as *p* < 0.05.

## Results

### A Cre-dependent rabies tracing approach for quantifying inputs to HPC subregion CA1

Genetically modified rabies tracing is a powerful tool for quantitative identification of direct circuit inputs to specific neuronal types (Wickersham et al., 2007; Sun et al., 2014, 2017b; DeNardo et al., 2015). In the present work, the approach was applied to accurately assess the relative strengths of different afferent sources for CA1 pyramidal neurons, which represent a major source of efferents in the HPC system. Viral tracing injections were made with the specific purpose of examining inputs directly reaching CA1 pyramidal neurons and with sensitivity to gradients in the strength of those inputs across the transverse/proximal-to-distal axis of CA1.

**Figure 1.**
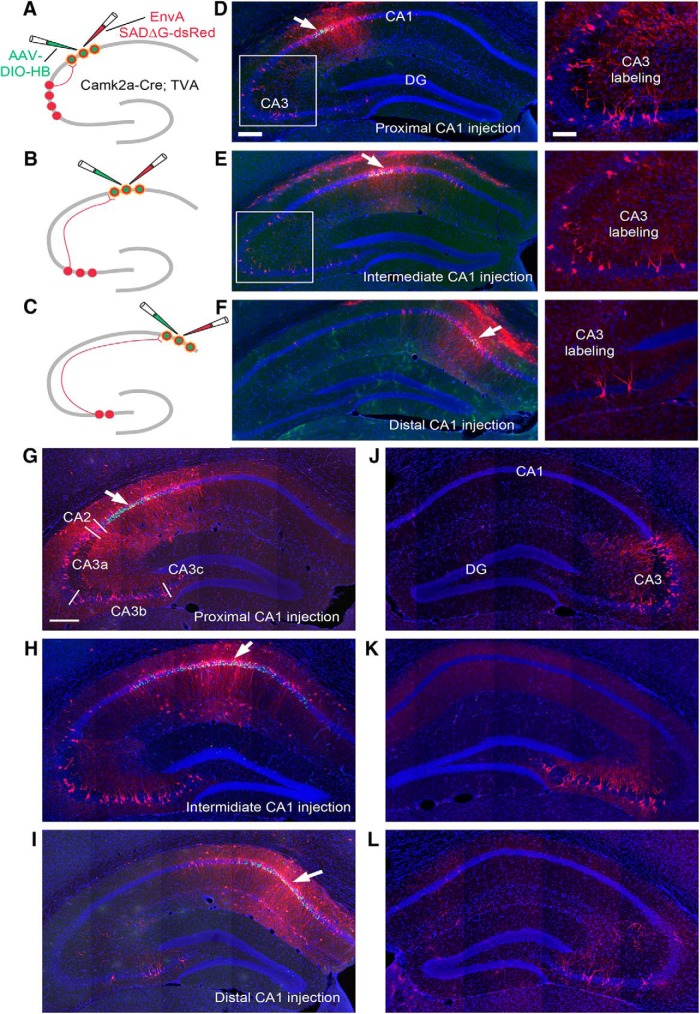
Topographic organization of CA3 to CA1 projections revealed through monosynaptic rabies tracing by specifically targeting CA1 pyramidal cells along the proximal-distal axis. ***A–C***, The schematic illustrates targeting proximal (***A***), intermediate (***B***), and distal (***C***) CA1 through a Cre-dependent rabies tracing system. A Cre-dependent AAV helper virus (AAV8-EF1a-DIO-HB) carrying histone GFP (hGFP) and rabies B19 glycoprotein (B19G) is injected into different CA1 subfields of the Camk2a-Cre; TVA mice. Thus, the AAV helper virus only expresses in CA1 excitatory pyramidal neurons. Three weeks later, an EnvA pseudotyped, glycoprotein deficient rabies virus encoding mCherry fluorescent protein (EnvA-SAD-ΔG-mCherry) is delivered into the same brain region of the AAV injection. Once rabies virus has infected the same group of neurons via the TVA receptor, it undergoes transcomplementation and spreads to the presynaptic partners of the targeted neurons and labels the presynaptic neurons with mCherry (shown in CA3). Note that some mCherry labeling of excitatory neurons around the injection is due to primary rabies infection, as pyramidal neurons expressing TVA can be directly infected by local injection of EnvA-ΔG rabies. Based on laminar position, putative inhibitory cells were labeled outside the pyramidal cell layer. ***D–F***, Examples show the injection sites (pointed by the arrows) in proximal (***D***), intermediate (***E***), and distal (***F***) CA1 by using iontophoretic injections. The corresponding CA3 labeling is shown in the enlarged panels on the right. Brain slices were sectioned coronally. DAPI staining is shown in blue, GFP expression of AAV helper virus is shown in green, and mCherry expression of rabies virus is shown in red. Neurons infected by both AAV and rabies, termed as starter neurons, appear in yellow. Scale bar on the left = 200 μm, right = 100 μm. ***G–I***, Examples show the injection sites (pointed by the arrows) in proximal (***G***), intermediate (***H***), and distal (***I***) CA1, by using pressure injections. CA3 labeling is shown in the same section. Three white bars in ***G*** indicate the divisions of CA3a, CA3b, and CA3c (subfields of CA3). The scale bar (200 μm) in ***G*** applies to ***G–L***. ***J–L***, Contralateral CA3 projections to proximal (***J***), intermediate (***K***), and distal (***L***) CA1. Also see Fig. 1-1.

We follow the basic nomenclature of Lorente De Nó (1934), used by later authors Ishizuka et al. (1990) and Naber et al. (Naber and Witter, 1998; Naber et al., 2001) to demarcate different HPC subregions along the transverse axis. Proximal (nearer to CA3/CA2) and distal (farther from CA3/CA2) CA1 sites designate positions along the transverse axis of CA1; proximal CA1 is located close to CA2, and distal CA1 is located toward the border of CA1/SUB (Fig. 1; Fig. 1-1). The CA3 region is subdivided as distal CA3/CA3a, middle CA3/CA3b, and proximal CA3/CA3c subregions, with CA3c nearest to the dentate gyrus. The midline of the fimbria separates CA3b and CA3a.

10.1523/ENEURO.0322-17.2018.f1-1 Extended Data Figure 1-1 Viral labeled neurons in CA1 injection sites. ***A–C***, Example images show the viral pressure injection sites in proximal CA1 (pCA1), intermediate CA1 (mCA1), and distal CA1 (dCA1), respectively. The scale bar (200 μm) applies to the top panels of ***A***, ***B***, and ***C***. Bottom panels are enlarged views of the white box region in their corresponding panels on top. Histone-tagged GFP expression of AAV helper virus is green, and mCherry expression of rabies virus is red. Double-labeled starter neurons appear yellow. The scale bar (50 μm) applies to the bottom panels of ***A***, ***B***, and ***C***. ***D***, A subiculum slice that is closest to the injection site in dCA1. The dotted line delineates the CA1 and subiculum border, which is defined by the abrupt change of pyramidal cell layer density. Scale bar = 200 μm. ***E***, Enlarged view of the white box in ***D***; rabies-labeled subiculum neurons are shown in red; there is no GFP expression (shown in green) of AAV helper virus in subiculum neurons. Scale bar = 100 μm. Download Figure 1-1, PDF file.

CA1 pyramidal neurons were targeted using Camk2a-Cre (T29) mice (Tsien et al., 1996), in which Cre expression is restricted to excitatory pyramidal cells. The monosynaptic rabies tracing method has been described in greater detail in our previous publication (Sun et al., 2014) and is described briefly here. The Camk2a-Cre mouse line is first crossed with a Cre-dependent TVA-expressing mouse line, LSL-R26^TVA-lacZ^ (Seidler et al., 2008), so that Cre-expressing neurons express TVA, thus restricting initial EnvA-ΔG rabies virus infection to Cre^+^ cells. Then, a Cre-dependent AAV (the helper virus AAV8-EF1α-FLEX-HB) with a coding sequence of rabies glycoprotein (RG) that is required for trans-synaptic rabies retrograde spread, as well as nuclear localized histone-GFP, is injected into dorsal CA1 of the double-transgenic mice (Camk2a-Cre; TVA mice). After the AAV injection, a pseudotyped deletion-mutant rabies virus encoding the red fluorescent protein mCherry (EnvA-SADΔG-mCherry rabies) is injected into the same location of the previous AAV injection. The EnvA pseudotyped ΔG-mCherry rabies enters the Cre^+^ and TVA^+^ neurons and undergoes genomic replication with mCherry expression. Using RGs expressed by the helper vector in the Cre^+^ cells, ΔG-mCherry rabies then undergoes transcomplementation (forming new infectious viral particles) and spreads to the presynaptic partners of the Cre^+^ starter neurons.

Using this method, we virally traced circuit connections to a small population of starter CA1 pyramidal cells in proximal (AP –2.06, ML –1.94, DV –1.51), intermediate (AP –2.06, ML –1.40, DV –1.35), and distal (AP –2.06, ML –0.85, DV –1.38) CA1 segments (Fig. 1*A–C*), using Camk2a-Cre; TVA mice. Three injection locations were chosen rather than two to better examine proximal-distal transitions and gradient connectivity. We used two methods to deliver helper AAV into different CA1 segments: Picospritzer pressure injection and iontophretic current injection (Fig. 1*D–I*; Fig. 1-1). Pressure injection produces broader injection sites with more starter neurons, whereas iontophretic injection produces injection sites that are spatially more restricted and prevents from any potential leak issues (e.g., leaks to CA2; Fig. 1 and Table 1). We verified that there are no starter neurons in CA2. There were several cases in our analysis of distal CA3 inputs to CA1 that showed a few CA2 labeled neurons, and we excluded these from further analysis. However, both methods produced similar input connection patterns for targeted neuron types. As all results are normalized by the number of the starter neurons specific to the injection site, all data were pooled for analysis.

**Table 1.** Quantitative strengths of specific input connections to proximal, intermediate, and distal CA1 pyramidal neurons

	Ipsilateral			Contralateral			Ipsilateral		Ipsilateral		Contralateral			Median Raphe	MS-DB
	CA3a	CA3b	CA3c	CA3a	CA3b	CA3c	LEC	MEC	Sub	Pre/Para Sub	pCA1	mCA1	dCA1		
Proximal CA1															
Average CSI	4.00	3.14	1.89	1.79	1.20	0.70	0.49	1.51	0.31	0.53	0.00	0.00	0.01	0.12	1.69
SE	0.55	0.31	0.23	0.13	0.11	0.14	0.08	0.24	0.04	0.11	0.00	0.00	0.01	0.01	0.23
*n*	6			5			6		8		5			5	5
Intermediate CA1															
Average CSI	2.61	2.32	1.06	1.15	1.03	0.51	0.62	0.87	0.55	0.28	0.01	0.04	0.13	0.05	0.88
SE	0.50	0.41	0.24	0.13	0.13	0.05	0.04	0.10	0.05	0.04	0.01	0.02	0.06	0.01	0.06
*n*	5			5			6		7		5			5	5
Distal CA1															
Average CSI	0.61	1.23	0.60	0.34	0.76	0.43	0.98	0.45	1.33	0.24	0.01	0.08	0.23	0.03	1.43
SE	0.10	0.13	0.08	0.12	0.19	0.14	0.11	0.07	0.27	0.06	0.00	0.02	0.04	0.01	0.17
*n*	5			5			6		7		5			5	6

Note that the input connection strength index (CSI) is defined as the ratio of the number of labeled presynaptic neurons in a specified structure versus the number of starter neurons. In each injection condition, 5–6 cases of Picospritzer-injected brains are taken for quantifications. The average number of starter neurons of Picospritzer injected cases are 126 ± 17 (proximal CA1 injections), 136 ± 32 (intermediate CA1 injections), and 80 ± 14 (distal CA1 injections). In addition, 4 cases of iontophoretic injected brains for each condition are included for quantification. The average numbers of starter neurons of iontophoretic injected cases are 21 ± 4 (proximal CA1 injections), 27 ± 5 (intermediate CA1 injections), and 26 ± 4 (distal CA1 injections). pCA1, proximal CA1; mCA1, intermediate CA1; dCA1, distal CA1; MS-DB, medial septum and diagonal band. See Table 1-1 for detailed statistical comparison results.

10.1523/ENEURO.0322-17.2018.f4-1 Extended Table 1-1 Download Table 1-1, DOCX file.

A total of 30 high-quality cases (no leak, robust labeling with unambiguous starter neurons limited to well-defined CA1 targeted sites; see Table 1 for specific numbers of cases used for detailed quantification) were used for quantitative analyses. Our first level of analysis was to visualize the injection site and ensure that double-labeled starter cells were restricted. Starter CA1 cells in brain sections were unambiguously identified by their GFP and mCherry expression from the helper AAV and ΔG-mCherry rabies genomes, respectively (Fig 1*D–I*; Fig. 1-1). The dependability of the method was verified by observance of labeled cells even in very distant structures such as the medial septal/diagonal band (MS-DB) area as well as reuniens thalamic nucleus and median raphe nucleus. These latter structures are known to project weakly to CA1 (Somogyi and Klausberger, 2005; Sun et al., 2014).

Because the number of starter cells and the number of direct presynaptic labeled cells in specified structures across the entire brain can be quantitatively determined, this approach allows for assessment of the relative strength in connectivity between subcomponents of HPC/EC circuits. We operationally define input connection strength index (CSI) as the ratio of the number of presynaptic neurons versus the number of starter neurons. This reflects the comparative number of presynaptic cells labeled by rabies in each region normalized by the number of infected CA1 pyramidal cells. Thus, this approach enables us to examine how the different sources of input to CA1 are distributed in different strengths onto excitatory neurons in different CA1 segments.

### Complementary topographic connectivity gradients of CA3 inputs and a bias to proximal CA1

Hippocampal CA1 receives abundant feed-forward excitation from CA3 pyramidal cells via their ipsilateral Schaffer collaterals and contralateral commissural fibers. In the present work, essentially all of the rabies-labeled CA3 cells were located in the stratum pyramidale (SP) and were morphologically and neurochemically confirmed to be excitatory neurons. Consistent with the previously described “flipped” topographic map of CA3 projections to CA1 along the transverse axis (Ishizuka et al., 1990; Li et al., 1994; Brivanlou et al., 2004), our rabies tracing data from different CA1 segments confirm previous anatomic observations that distal CA3a neurons project most strongly to nearby proximal CA1 (Fig. 1*A*, *D*, *G*, *J*). In a complementary fashion, distal CA1 receives its strongest CA3 input from the more proximal CA3b (Fig. 1*C*, *F*, *I*, *L*) and about equally strong input connections from CA3a and CA3c. Intermediate CA1 inputs from CA3 were more distributed along the transverse axis of CA3 (Fig. 1*B*, *E*, *H*, *K*), in line with prior observations that connectivity differences across the transverse axis follow a gradient as opposed to having sharp boundaries.

We extended these earlier qualitative anatomic observations with quantitative evaluation of CA3 input strengths and found unexpected biases in overall CA3 inputs to CA1 that follow the transverse axis. Proximal CA1 receives >3-fold more overall CA3 inputs than those received by distal CA1 (Table 1; Fig. 6*A*, *B*). This bias was observed for both ipsilateral and more distant and less strong contralateral CA3a-c connectivity to CA1. Contralateral CA3 inputs to CA1 also followed the complementary topographic connectivity gradient seen for the aforementioned ipsilateral inputs. Thus, the sum total of CA3 input to CA1 is strongly biased to proximal CA1, becoming progressively weaker along the proximal-distal CA1 axis (Figs. 1 and 6*A*, *B*). For ipsilateral CA3 inputs, proximal CA1 receives very strong overall CA3 inputs, with its strongest input from CA3a (CSI 4.00 ± 0.55), followed by CA3b (3.14 ± 0.31) and CA3c (1.89 ± 0.23). Intermediate CA1 appears to receive weaker CA3 inputs relative to proximal CA1, with CSIs of CA3a, CA3b, and CA3c being 2.61 ± 0.50, 2.32 ± 0.41, and 1.06 ± 0.24, respectively. Distal CA1 receives the relatively weakest CA3 input, with CSIs of CA3a, CA3b, and CA3c being 0.61 ± 0.10, 1.23 ± 0.13, and 0.60 ± 0.08, respectively. Compared with other CA3 subregions, CA3c provides the least number of inputs to CA1 (Fig. 6*A*; Table 1). See Table 1-1 for detailed statistical comparison results. Our finding that proximal CA1 receives much stronger overall CA3 inputs than distal CA1 suggests that CA3-associated Schaffer collateral and contralateral commissural inputs have much stronger influence on neural activity of proximal CA1 than distal CA1 in dorsal hippocampus.

### Contralateral CA1, median raphe, and MS-DB inputs to CA1 pyramidal neurons

Compared with contralateral CA3 inputs, we find that inputs from contralateral CA1 are weak and are mostly from contralateral distal CA1 excitatory neurons (overall CSI of contralateral distal CA1: 0.32). Distal CA1 inputs to contralateral CA1 appear to have stronger interhemispheric/contralateral CA1 connections than the proximal CA1 (CSI 0.01; Fig. 1*J–L*; Table 1). We did not quantify local excitatory connections in CA1 ipsilateral to the injection sites, as labeled CA1 excitatory cells around the injection site could not be distinguished as primarily versus secondarily infected by rabies in Camk2a-Cre; TVA cases. For distant brain structures, the median raphe sends relatively stronger input to proximal CA1 (CSI 0.12 ± 0.01) than distal CA1 (0.03 ± 0.01). The MS-DB inputs show moderate strengths to both proximal and distal CA1 (CSI 1.69 ± 0.23 and 1.43 ± 0.17), but the MS-DB input to intermediate CA1 is weaker (CSI 0.88 ± 0.06; Table 1). See Table 1-1 for detailed statistical comparison results.

### Topography of MEC/LEC inputs to CA1 pyramidal neurons

**Figure 2.**
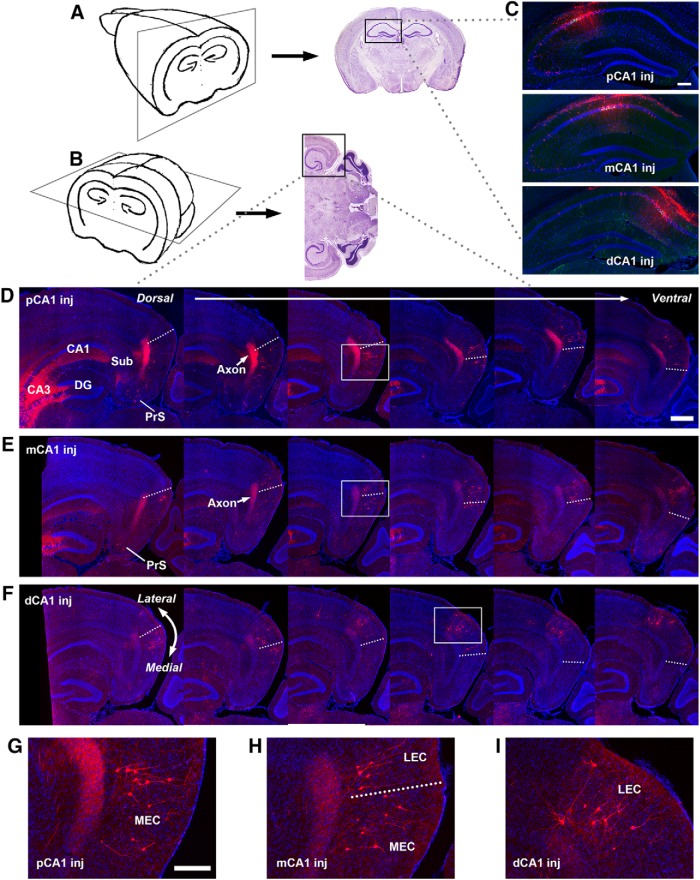
Topographic organization of lateral and medial entorhinal cortex inputs to different CA1 subfields. ***A***, ***B***, Schematic illustration of the combined coronal/horizontal sectioning approach. The rostral portion of the brain was sectioned in the coronal plane to identify the injection site in CA1 (***A***), while the caudal portion was sectioned in the horizontal plane to delineate lateral versus medial EC (***B***). ***C***, Example injection sites in proximal CA1 (pCA1), intermediate CA1 (mCA1), and distal CA1 (dCA1), respectively. Scale bar = 200 μm. ***D–F***, Retrogradely labeled EC neurons following rabies tracing in proximal CA1 (***D***), intermediate CA1 (***E***), and distal CA1 (***F***), respectively. Images from the left to right are organized from the dorsal to ventral. Rabies-labeled neurons are red. DAPI staining is blue. The arrow points to the prominent axons terminating in the deeper layers of the entorhinal cortex. These axons likely come from rabies-infected cells around the injection site in CA1. Dashed lines indicate the MEC and LEC border. The scale bar (500 μm) applies to all the other panels. ***G***, Enlarged image of the white box region in ***D***. ***H***, Enlarged image of the white box region in ***E***. ***I***, Enlarged image of the white box region in ***F***. The scale bar (200 μm) applies to ***G–I***.

In comparison with the complementary topographic connectivity gradients of CA3a-c, opposing connectivity gradients occur for the MEC (strong-to-weak) and the LEC (weak-to-strong) inputs to CA1 pyramidal neurons along CA1 transverse axis. To better identify the laminar structure of the EC, we used a combined coronal/horizontal cutting strategy to section the brain (Steward, 1976; Fig. 2*A–C*). Our rabies tracing shows that EC inputs to CA1 are almost exclusively ipsilateral. MEC forms strong inputs to proximal CA1, which systematically diminishes in input strength along the CA1 proximal-distal axis (Figs. 2*D–I* and 6*C*; Table 1). Direct MEC to proximal CA1 inputs have a CSI of 1.51 ± 0.24, which is about 3-fold greater than the LEC counterpart (CSI 0.49 ± 0.08; Figs. 2*D*, *G*, and 6*C*). MEC also has relatively stronger input to intermediate CA1 than LEC (CSI 0.87 ± 0.10 vs. 0.62 ± 0.04; Figs. 2*E*, *H*, and 6*C*). By contrast, LEC input strengths show an opposing trend along the transverse axis. Distal CA1 receives ∼2-fold more input from LEC than MEC, with the CSIs of LEC and MEC being 0.98 ± 0.11 and 0.45 ± 0.07, respectively (Figs. 2*F*, *I*, and 6*C*; Table 1). Overall, MEC prevails in the EC projection into CA1, with 35% greater summed input than LEC.

As CA1 neurons provide direct projections into EC deep layers (Tamamaki and Nojyo, 1995), prominent axons originating from the rabies labeled CA1 neurons around the injection site terminate in layers V and VI of EC (Fig. 2*D–F*). Similar to the topography of the EC projections to CA1, the axon termination zones shift from the medial to the lateral of EC along with the injection sites shifting from proximal CA1 to distal CA1. In confirmation, the region of rabies-labeled entorhinal neurons located in EC matches the region of CA1 incoming EC-projecting axons. This is supported by previous studies reporting that the origin and termination of CA1 to EC projections are in register (Tamamaki and Nojyo, 1995; Naber et al., 2001).

**Figure 3.**
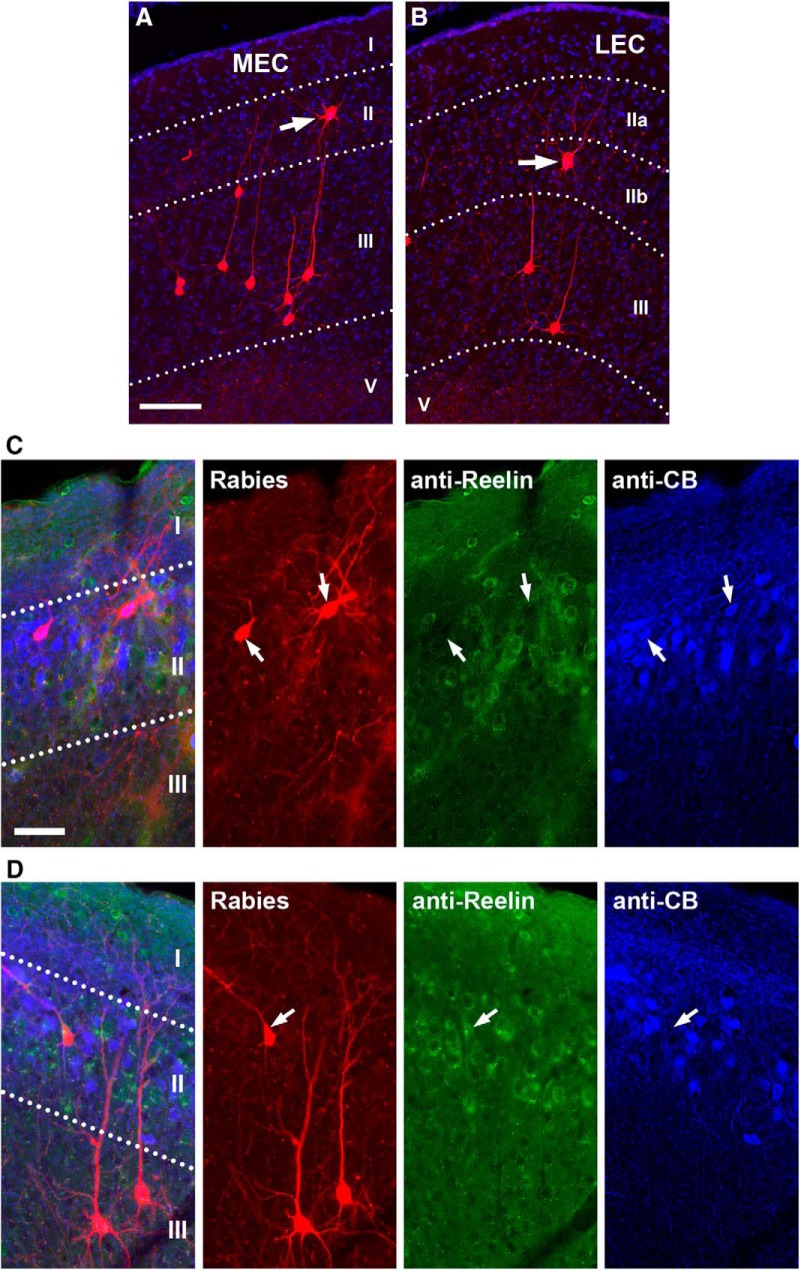
A moderate portion of layer II EC neurons project directly to hippocampal CA1. ***A***, Rabies-labeled neurons in the medial entorhinal cortex (MEC) contain both layer III pyramidal cells and layer II cells. The white arrow points to a layer II cell. The scale bar (100 μm) applies to both ***A*** and ***B***. ***B***, Rabies-labeled neurons in the lateral entorhinal cortex (LEC) also contain layer III pyramidal cells and layer II cells. ***C***, The example rabies-labeled MEC layer II cells are calbindin (CB) immunopositive, but reelin negative. ***D***, The example rabies-labeled MEC layer II cell is reelin immunopositive, but CB negative. Scale bar in ***C*** = 100 µm, applies to both ***C*** and ***D***.

In addition, we consider “direct” versus “indirect” pathways for EC inputs to CA1. CA1 neurons receive direct input from EC layer III, whereas EC layer II neurons project to CA1 indirectly via the dentate gyrus or CA3 (as discussed above). This earlier result is consistent with the current rabies-labeling results in that the majority of EC inputs to CA1 pyramidal neurons arise from layer III putative pyramidal cells (Fig. 2*G–I*). However, we did find that ∼15% of the rabies-labeled EC neurons arose from layer II of the entorhinal cortex (Fig. 3*A*, *B*). Double immunostaining against CB and reelin in selected sections (18 neurons pooled from 6 different cases; Fig. 3*C*, *D*) showed that 50% of rabies-labeled EC layer II neurons (including both MEC and LEC) are immunopositive only for CB and 22% of them are immunopositive only for reelin. The remaining 28% cells are positive for neither. This is likely due to immunostaining sensitivity, as we included only robustly stained cells for quantification. Thus these CA1-projecting EC layer II cells might include some CB^+^ cells that also innervate SLM-interneurons in CA1 (Kitamura et al., 2014).

### A topographic gradient of SUB inputs to CA1 with a strong bias to distal CA1

The CA1 projection to the subiculum has a mirrored topography, in that distal CA1 projects to proximal subiculum, and proximal CA1 projects to distal subiculum (Amaral et al., 1991; Amaral, 1993). The canonical circuit connections of the hippocampal formation are feed-forward in terms of the directionality of information flow. Within this framework, SUB is viewed as a major output subregion of HPC (O’Mara et al., 2001; Witter, 2006; O’Mara, 2005; Cenquizca and Swanson, 2007). However, accumulating evidence prompts an update of this traditional view of unidirectional projection between hippocampal CA1 and SUB. Alongside previous studies in other species (Berger et al., 1980; Finch et al., 1983; Köhler, 1985; Harris and Stewart, 2001; Shao and Dudek, 2005), we recently characterized noncanonical back-projections from SUB to CA1 in the mouse (Sun et al., 2014; Xu et al., 2016). This previously unappreciated back-projection pathway could potentially serve to modulate hippocampal information processing (Jackson et al., 2014; Craig and McBain, 2015). As the feed-forward CA1 projection to the SUB has a strong mirrored topography, it is important to examine whether SUB shows a specific transverse topography in its CA1 projections.

**Figure 4.**
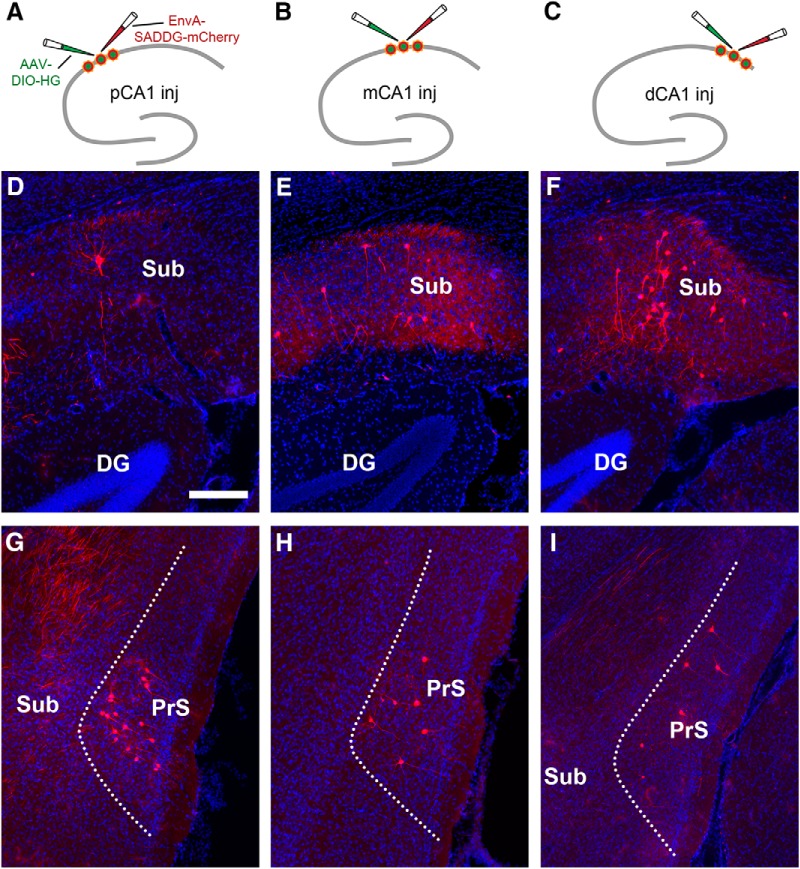
The subiculum strongly projects to distal CA1 while the presubiculum strongly projects to pCA1, thus showing opposing proximal-distal connectivity gradients. ***A–C***, Schematic illustrations rabies tracing from proximal CA1 (pCA1, ***A***), intermediate CA1 (mCA1, ***B***), and distal CA1 (dCA1, ***C***), respectively. ***D–F***, Rabies labeled CA1-projecting subicular neurons in example cases following the injection in pCA1 (***D***), mCA1 (***E***), and dCA1 (***F***), respectively. The scale bar (200 μm) in ***D*** applies to all other panels. ***G–I***, Rabies-labeled CA1-projecting presubicular neurons in example cases following the injection in pCA1 (***G***), mCA1 (***H***), and dCA1 (***I***), respectively. Also see Fig. 4-1.

Our tracing data show that a significant number of SUB cells are retrogradely labeled by rabies tracing from CA1 pyramidal neurons in dorsal HPC (Fig. 4*A–F*; Fig. 4-1). All labeled neurons are found in dorsal SUB ipsilaterally. Further, no rabies-labeled cells are seen in ventral SUB (Fig. 4-1). The labeled neurons appear morphologically to include both excitatory neurons and inhibitory interneurons (also see Sun et al., 2014), and they are predominantly located in the pyramidal cell layer, with average percentages of laminar distributions of 92%, 4%, and 4% for pyramidal cell, polymorphic, and molecular layers, respectively (data measured from 5 cases, Fig. 6*E*).

10.1523/ENEURO.0322-17.2018.f4-1 Extended Data Figure 4-1 Rabies tracing reveals noncanonical subiculum complex inputs to different CA1 subfields. ***A–D***, Labeling of CA1-projecting subicular neurons in dorsal subiculum (dSub) in different sections along the rostral-caudal axis. The scale bar (200 μm) in ***C*** applies to all other panels. ***E***, Rabies-labeled neurons in the presubiculum (PrS) and parasubiculum indicate that these neurons directly project to proximal CA1. ***F***, No labeled neurons in ventral subiculum (vSub) indicate no direct input from vSub to distal CA1. ***G–L*** and ***M–R*** are formatted similarly to ***A–F***, for rabies tracing from intermediate and distal CA1. Download Figure 4-1, PDF file.

Similar to the topographic connectivity gradient of LEC input, there is a weak-to-strong SUB input strength gradient along the proximal-distal axis of CA1 (Figs. 4*D–F* and 6*D*; Table 1). However, the quantification enabled by the rabies tracing technique reveals a strong bias within the gradient. Distal CA1 (CSI 1.33 ± 0.27) receives >2.5- and 4-fold more input from SUB compared, respectively, with intermediate CA1 (CSI 0.55 ± 0.05) and proximal CA1 (CSI 0.31 ± 0.04; Fig. 6*D*; Table 1). Surprisingly, the connection strength of SUB back-projections to distal CA1 is comparable quantitatively to those of EC and MS-DB inputs (Table 1). Thus, SUB sends a major projection to CA1 neurons in a highly topographic manner. The sharpness of the gradient yields a SUB to distal CA1 connectivity strength that surpasses that of the direct connections from LEC to distal CA1 (Fig. 6*C*, *D*; Table 1).

### Presubiculum and parasubiculum provide noncanonical direct inputs to CA1

**Figure 5.**
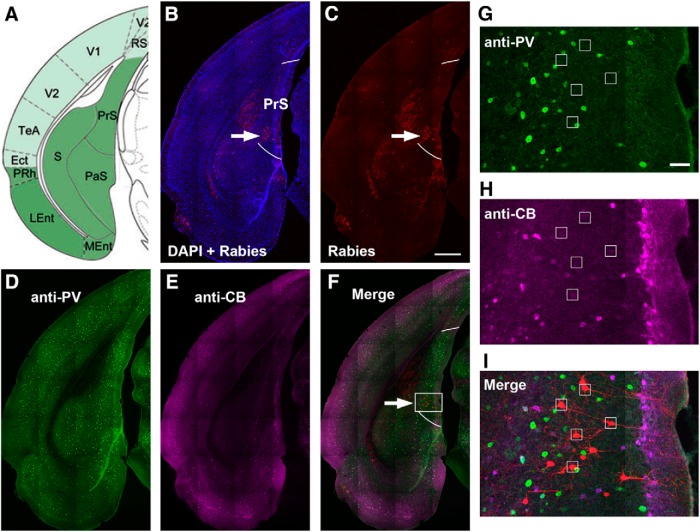
Rabies-labeled CA1-projecting neurons in immunochemically delineated presubiculum are mostly excitatory neurons. ***A***, The mouse atlas image shows the anatomic location of the presubiculum (PrS). ***B***, ***C***, A brain section image corresponding to the atlas image. Rabies-labeled neurons appear in the presubiculum (indicated by the white arrow) after CA1 virus injection. DAPI staining is blue. Two white lines delineate the region of PrS per PV and CB immunochemical staining. The scale bar (500 μm) applies to ***B–F***. ***D–F***, Images of parvalbumin (PV) immunostaining (green, ***D***), calbindin-D28k (CB) immunostaining (magenta, ***E***), and a merged image (***F***). White arrow points to the region with rabies-labeled neurons, which has strong PV immunoreactivity and weak calbindin immunoreactivity. The PV and CB immunoreactivity features allow for delineation of PrS (Fujimaru and Kosaka, 1996; Fujise et al., 1995). ***G–I***, Enlarged images of the white box region in ***F*** show PV staining (***G***), CB staining (***H***), and rabies-labeled neurons (red) in the merged image (***I***). Small white squares locate rabies-labeled neurons that are not positive for PV or CB staining. The scale bar (100 μm) in ***G*** applies to ***G–I***.

We also observed a group of CA1-projecting neurons originating from other parts of the subicular complex, the presubiculum and parasubiculum. These regions were immunochemically delineated, as they are characterized by strong CB staining in layer II and strong PV staining in deep layers, which distinguishes from patchy-like CB staining in EC (Fujise et al., 1995; Fujimaru and Kosaka, 1996). Most rabies-labeled neurons were located in layer III of the presubiculum and parasubiculum (Figs. 2*D–F*, 4*G–I*, and 5). Based on their morphology and lack of CB or PV immunoreactivity, the labeled neurons are likely pyramidal cells (Fig. 5). The tracing data demonstrate that CA1 excitatory neurons are innervated directly by the presubiculum and parasubiculum, beyond the subiculum.

Presubiculum and parasubiculum inputs to CA1 are quantitatively weaker than SUB to CA1 inputs, and their input strengths gradually weaken along the proximal/distal axis. Overall, proximal CA1 (CSI 0.53 ± 0.11) receives stronger inputs from the presubiculum/parasubiculum, with progressively weaker inputs to intermediate CA1 (CSI 0.28 ± 0.04) and distal CA1 (CSI 0.24 ± 0.06; Fig. 4*G–I*; Fig. 4-1*E*, *K*, *Q*; Fig. 2*D–F*; Table 1). As the presubiculum/parasubiculum contains abundant functional cell types including grid cells, head-direction cells, and border cells as measured and defined physiologically (Boccara et al., 2010), our findings provide an anatomic circuit basis for these functional cell types in the presubiculum/parasubiculum to directly influence proximal CA1 place cells, which exhibit stronger place-specific modulation.

### Functional validation of SUB topographic connectivity gradients

The strength and shape of the noncanonical SUB input connectivity gradient compelled us to examine functional connection topography using fast VSD imaging of neural activity and laser photostimulation via glutamate uncaging in brain slice preparations (Fig. 7*A*). Given that intrahippocampal connections are dense and largely intact in some planes of sections oriented perpendicularly to the long axis of the hippocampus, functional examination of topographic SUB-CA1 connections were performed in living brain slice preparations. The notion of a topographic projection from SUB to CA1 is supported by previous slice mapping experiments (Shao and Dudek, 2005). In calibrated conditions, laser photostimulation via glutamate uncaging offers spatially restricted neuronal activation, and only neurons located close to photostimulation sites fire action potentials (Olivas et al., 2012; Shi et al., 2014). The observed ensemble VSD signals are closely related to membrane potential depolarization of individual neurons (Xu et al., 2010a; Shi et al., 2014). The combined stimulation and imaging approach allows us to map direct projections from different SUB regions to their targeted CA1 locations by VSD imaging of evoked activation.

**Figure 6.**
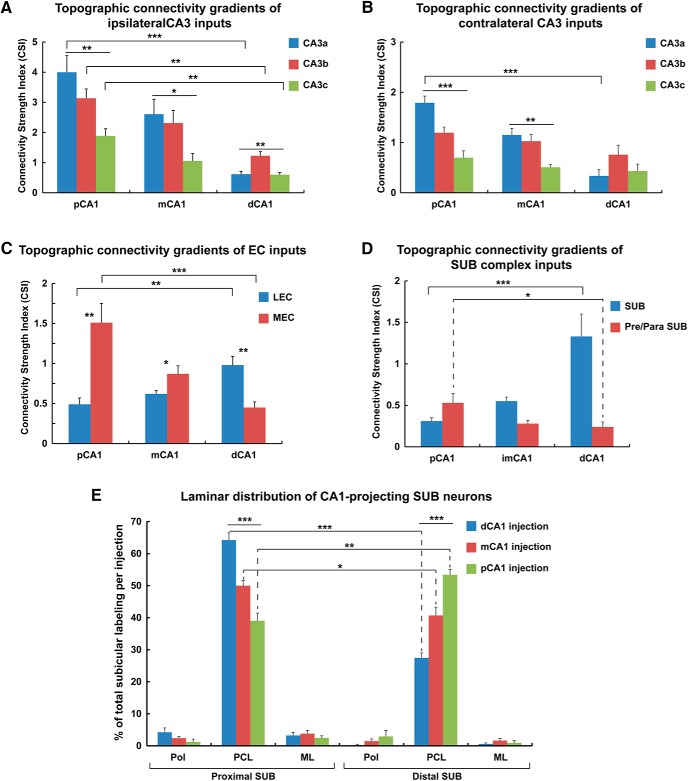
Quantitative analyses of proximal-distal connectivity gradients of canonical CA3/EC and noncanonical subiculum complex inputs to CA1 subfields. ***A***, A quantitative summary shows input strength differences of canonical CA3 subregion (CA3a, CA3b, and CA3c) inputs to proximal CA1, intermediate CA1, and distal CA1, respectively. The data are plotted with the values of the connectivity strength index (CSI), defined as the number of presynaptic neurons in a specific brain region divided by the number of starter neurons in the injections site. The data show complementary gradually significant decreases from strong CA3a–c connectivity to proximal CA1 to progressively weaker connectivity for CA3a–c to intermediate CA1 and distal CA1. These complementary decreases show systematically strong-to-weak connectivity along the proximal-distal axis of CA1. Group comparisons performed using one-way ANOVA with Tukey *post hoc* tests. The data are presented as mean ± SE; *, **, and *** indicate the statistical significance levels of *p* < 0.05, 0.01, and 0.001, respectively. ***B***, A quantitative summary shows gradient-similar complementary input strength differences of contralateral CA3 (CA3a, CA3b, and CA3c) inputs to proximal CA1, intermediate CA1, and distal CA1, respectively. ***C***, Opposing connectivity gradients seen for canonical medial entorhinal (MEC) and lateral entorhinal cortex (LEC) inputs to CA1. A quantitative summary shows input strength differences of LEC (gradually increasing along the proximal-distal axis) versus MEC (gradually decreasing along the proximal distal axis) to proximal CA1, intermediate CA1, and distal CA1, respectively. The data are presented as mean ± SE; *, **, and *** indicate the statistical significance levels of *p* < 0.05, 0.01, and 0.001 respectively. ***D***, Opposing connectivity gradients seen for noncanonical subiculum (increase along the proximal-distal axis) to CA1 versus presubiculum (decrease along the proximal-distal axis) to CA1. A quantitative summary of connectivity strengths of the subiculum versus the presubiculum/parasubiculum to proximal CA1, intermediate CA1, and distal CA1 is shown. The data are presented as mean ± SE; * and *** indicate the statistical significance levels of *p* < 0.05 and 0.001, respectively. ***E***, Laminar distribution of CA1-projecting subicular neurons after rabies tracing from distal CA1, intermediate CA1, and proximal CA1. The bar graph is plotted as the percentage of total labeled subicular neurons in each case. Pol, polymorphic layer; PCL, pyramidal cell layer; ML, molecular layer. The data are presented as mean ± SE; * and *** indicate the statistical significance levels of *p* < 0.05 and 0.001, respectively. See Table 1-1 for detailed statistical comparison results.

**Figure 7.**
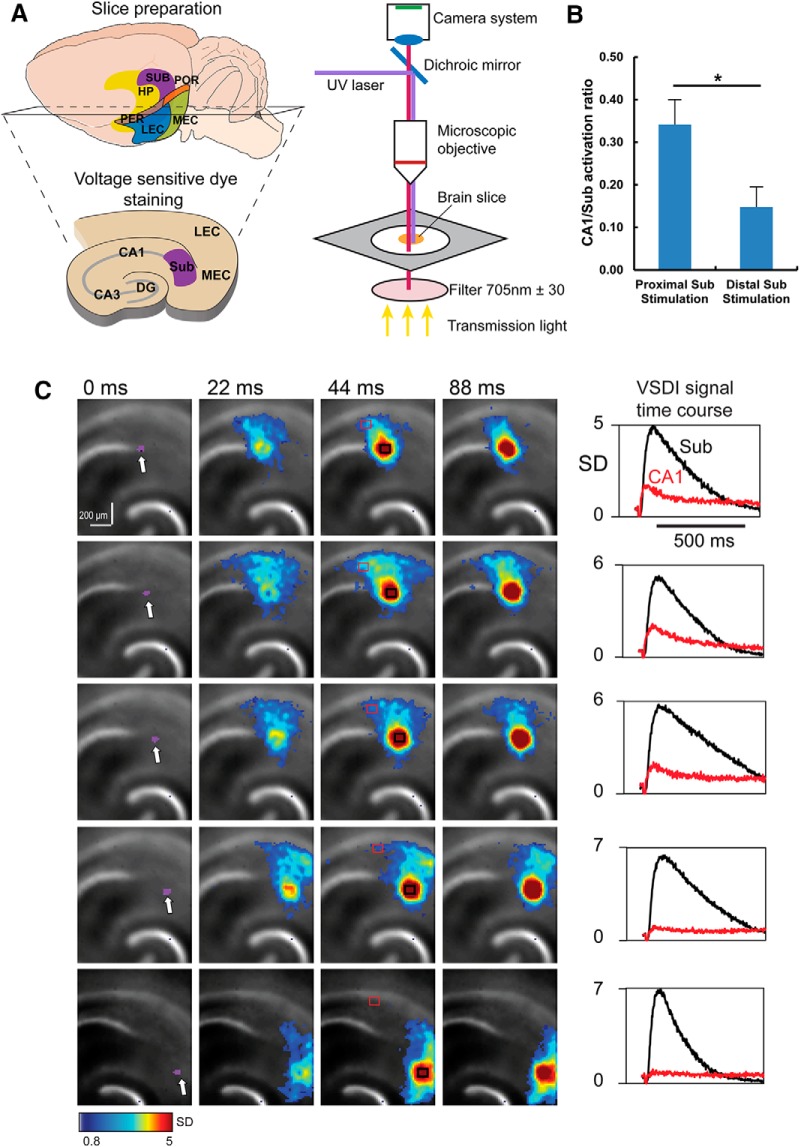
Functional circuit mapping with fast VSD imaging of neural activity validates the topographic organization of the subiculum to CA1 back-projections. ***A***, Schematic of slice preparation for VSD imaging. ***B***, A summary plot shows that the average ratio of CA1/subiculum mean activity decreases significantly (*, p = 0.046) when the photostimulation occurs in the more proximal subiculum versus the more distal subiculum. The VSD data physiologically validate the topographic organization of the subiculum to CA1 back-projections. ***C***, Time series data from VSD imaging after photostimulation-evoked neural activation in different subiculum subfields. The purple dots (laser stimulation artifact) at 0 ms indicate the photostimulation site. The stimulation site in the subiculum shifts gradually away from the CA1/subiculum border from the top to the bottom of the panels. Color-coded activity is superimposed on the background slice image. The color scale codes VSD signal amplitude expressed as SD multiples above the mean baseline. The stronger activation is indicated by the warmer color. On the right, time course plots of VSD imaging (VSDI) signal from the regions of interest (subiculum and CA1) indicated by the black and red rectangles in the corresponding image frame on the left, respectively, starting from the baseline of 22 ms before the photostimulation onset.

**Figure 8.**
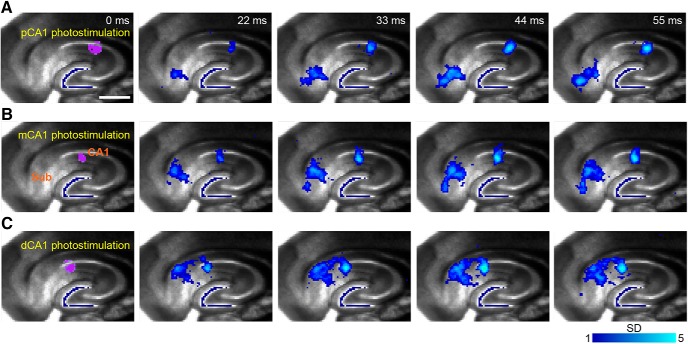
Functional circuit mapping with fast VSD imaging of neural activity demonstrates a strong, discrete topographic organization of CA1 to subiculum projections. ***A–C***, Spatially restricted photostimulation in proximal CA1 (pCA1, ***A***), intermediate CA1 (mCA1, ***B***), and distal CA1 (dCA1, ***C***) evokes discrete activation in the distal, intermediate, and proximal subiculum, respectively. Each panel shows time series data of VSD imaging of photostimulation-evoked neural activation in different hippocampal CA1 subfields. The purple dots (laser stimulation artifact) at 0 ms indicate the photostimulation site. Color-coded activity is superimposed on the background slice image. The color scale codes VSD signal amplitude expressed as SD multiples above the mean baseline. The stronger activation is indicated by a brighter color. Scale bar in ***A*** = 500 μm applies to all the panels.

We found that CA1 responses reliably followed stimulation of the SUB, particularly from proximal SUB, with locations within 200–400 μm from the CA1/SUB border. Spatially restricted photostimulation in SUB first initiates local activation, and then excitatory signals propagate back toward distal CA1 (Fig. 7*C*). These observations were consistent across 5 different slice experiments using 4 different animals. The average peak VSD response latencies of SUB and CA1 signals were 40.8 ± 4.1 and 78.2 ± 3.8 ms, respectively, after SUB photostimulation (*n* = 7 samples, *p* = 0.0007). CA1 activation in response to SUB stimulation decreases rapidly with further distance from the border (Fig. 7*B*, *C*). SUB stimulation more strongly impacts distal CA1 compared with proximal CA1. Thus, these physiologic data show excellent correspondence to rabies tracing-based anatomic connectivity strengths along the transverse axis. As another means of validating physiologic/functional circuit mapping, strong topographic CA1-SUB projections are revealed using the same approach; as expected from previous anatomic tracing studies, the CA1 stimulation and SUB activation sites show a discrete mirrored topography relative to the CA1/SUB border (Fig. 8). Together, these data indicate strong interactions between distal CA1 and SUB and suggest that noncanonical subiculum inputs cotrack the weak-to-strong LEC inputs along the transverse axis.

## Discussion

Using genetically modified rabies tracing, we quantitatively mapped the strengths of multiple distinct inputs directly synapsing on dorsal CA1 pyramidal neurons. Systematic variation in the placement of viral tracing injections along CA1 transverse axis enabled us to compare connectivity strengths across two circuits that divide the canonical trisynaptic pathway. Further, the same approach permitted evaluation of the specificity of noncanonical CA1 inputs to positions along the transverse axis.

Detailed analysis of CA1 pyramidal neuron inputs indicates strong connectivity gradients for the CA3 and EC neuron populations supplying afferents to CA1. This CA3/EC and CA1 connectivity patterns have been the basis for a major division of the canonical HPC pathway into semi-overlapping, but distinct, circuits and currently provide an important framework for neurophysiological investigations throughout all HPC and EC subregions. Although the canonical HPC circuit has been established in prior work, it has not been quantified until now. Our quantification indicates that the division is much more complex than has previously been appreciated. First, the sum total of CA3 and EC output to CA1 is clearly biased to proximal CA1. This distinction is particularly striking in the case of distal CA3 inputs and is further complemented by the previously known bias of EC inputs to distal CA3 as opposed to proximal CA3 (Sun et al., 2017a). Second, recently discovered, noncanonical SUB inputs to CA1 are found to be extensive to CA1 pyramidal neurons, and this projection is heavily biased toward distal CA1. The functional significance of this anatomic organization was confirmed by physiologic imaging work showing that stimulation of SUB most powerfully excites distal as opposed to proximal CA1 neurons. Further evidence of the impact of this input was found in prior work showing that SUB impacts theta-frequency field potential rhythms and spiking in CA1 (Jackson et al., 2014; Craig and McBain, 2015). Finally, we were able to clearly identify a significant population of presubiculum and parasubiculum neurons with direct projections to CA1 and show that these inputs are biased to proximal CA1.

To date, the differential functions and neurophysiology of MEC and LEC have provided the main basis for theory and experimentation addressing the transverse-axis division of the canonical HPC circuit (Witter et al., 1989, 1990; Knierim, 2006; Witter, 2007; Henriksen et al., 2010; Igarashi et al., 2014; Lee et al., 2015; Sun et al., 2017a). It has been suggested that MEC serves to generate a map of position relative to the hierarchically highest frame of reference for spatial tuning, and the boundaries of the observable environment (Neunuebel et al., 2013; Knierim et al., 2014). LEC is theorized to encode information in more local spatial frames of reference. Specifically, both HPC and EC neurophysiological functions are considered from the perspective of different forms of spatial tuning present in neurons of each area. Neurons throughout the HPC proper (DG, CA3, CA1, and even SUB) often exhibit robust place-specific firing, whereas MEC neuron subpopulations exhibit grid-patterned spatial tuning, sensitivity to specific environmental borders, or tuning to head orientation. From this perspective, the most striking difference between MEC and LEC is the relatively poorer spatial tuning observed in LEC and the presence of tuning of LEC neurons to objects within an environment. These differences in turn form the basis for interpretation of differences in spatial tuning specificity and adaptability of spatial tuning that are apparent by comparing neurons recorded along different sites of the transverse axes of CA1 and CA3.

While not challenging the importance of the canonical organization of the HPC circuit, our new data call for a revision of this perspective to include noncanonical inputs and will guide future experiments. In this respect, the most notable novel concepts adding to prior conceptions include (1) the idea that proximal CA1 is likely to be much more strongly influenced by CA3 than distal CA1; (2) the observance that proximal CA1 may be influenced by both presubiculum and parasubiculum; and (3) the idea that distal CA1 is likely to be more strongly influenced by SUB inputs than by LEC inputs. The more specific consequences of this change in perspective as they relate to encoding of spatial information are detailed below.

The current data indicate that proximal, relative to distal, CA1 is not only biased toward distal CA3 and MEC inputs, but is also subject to greater influence by CA3 and EC overall. Previous studies have shown that both distal CA3 and MEC ensemble spatial firing patterns can be described as being highly coherent after alterations in the shape and/or appearance of environmental boundaries. MEC grid cell pairs within the same module maintain consistent spacing between grid firing nodes across very different environments, yielding the original description of MEC grid cell networks as a “universal map” (Hafting et al., 2005). CA3 populations, particularly those in distal regions that innervate proximal CA1, exhibit strong tendencies for retention of the relative spatial distributions of their individual firing fields, both when conflicts in local versus distal cues are implemented and when two different foraging arenas are similar enough in their shape and layout of prominent boundary cues (Lee et al., 2015; Lu et al., 2015). Based on the present data, one would predict that proximal CA1 neurons, clearly dominated by these two inputs, would exhibit similar properties. Adding to this proposition, proximal CA1 also receives a greater density of presubiculum inputs, and temporary inactivation of presubiculum is known to degrade place-specific firing of CA1 neurons in familiar environments (Bett et al., 2013). Thus, presubiculum and parasubiculum may significantly contribute to place-specific firing in proximal CA1. Based on these considerations, a more specific prediction is that partial remapping of place-specific firing fields among a population of proximal CA1 neurons should be low. The bias of proximal CA1 outputs to MEC would also be viewed as supporting a computation reflecting correspondence between MEC grid cell network and distal CA3 place cell network which map position within the full environment. It remains to be determined whether such a result accompanies the known increase in spatial information and sparsity seen for proximal compared to distal CA1 neurons (Henriksen et al., 2010).

The unexpectedly strong presence of SUB inputs to distal CA1 opens new questions concerning the function of this subregion of CA1 and may explain several previous findings concerning the impact of SUB lesions on navigation and modulation of spatially tuned firing of CA1 populations in open arenas. Prior work comparing SUB-only versus HPC lesions established that SUB is critical for performance on the Morris water navigational task and, further, suggested that SUB and CA1 could potentially cooperate in guiding navigational behavior (Morris et al., 1990). Compared with CA1, spatial tuning of firing activity for SUB neurons is relatively weak and noisy, at least for recordings made in the context of open arena foraging (Sharp, 1999; Sharp and Green, 1994; Lever et al., 2009; Stewart et al., 2014; Olson et al., 2017). Yet prior work has established that the rough spatially tuned firing of SUB neurons is sensitive to head orientation, a result that may explain the recent demonstration of orientation sensitivity for open-field place-specific firing in a subpopulation of CA1 neurons (Acharya et al., 2016). The observed inputs to CA1 from presubiculum may also play a critical role in direction-selectivity of CA1 neurons in that such neurons often exhibit a high degree of selectivity to head orientation relative to the larger environment (Taube et al., 1990).

Compared with CA1, SUB neurons are known to exhibit greater generalization across environments differing in shape or scale but having similarity in the layout of distal visual cues. This property of SUB networks could potentially explain the propensity for CA1 populations to exhibit highly similar spatial firing patterns under circumstances in which animals explore two environments sharing shape, alignment, and spatial distribution of boundary cues (Skaggs and McNaughton, 1998; Spiers et al., 2015; Grieves et al., 2016). Notably, this phenomenon has direct impact in promoting learning of nonspatial associations (Morris et al., 1990; Grieves et al., 2016) and is consistent with recent findings revealing a role for proximal SUB, proximal CA3, and LEC in memory for exposure to specific odors (Nakamura et al., 2013; Ku et al., 2017). Finally, the organization of CA1 place-specific firing according to the reference frame of the observable environmental boundaries may be enhanced by SUB “boundary vector cells” that encode specific environmental borders (Lever et al., 2009).

In summary, the observed contrast in afferent sources for distal versus proximal CA1 pyramidal neurons indicates that the transverse-axis division of the canonical trisynaptic pathway must be considered as one that reflects a bias toward MEC and distal CA3 influences on the one hand (proximal CA1), and a bias toward SUB and LEC influences on the other (distal CA1). Proximal CA1 may function to precisely and stably encode position relative to the boundaries of the observable environment. Here, coherent spatial patterns among the firing fields of its neuron ensembles would be found whenever alterations in environmental boundaries lie below threshold for discriminating two environments as distinct contexts. Distal CA1 is biased to inputs from SUB that reflect the orientation and location of specific path components of different paths within a given environment. LEC contributions to distal CA1 may provide more local information concerning the presence and locations of specific sensory content such as objects. In line with the present anatomic data, recent theory (Eichenbaum et al., 2007; Neunuebel et al., 2013; Knierim et al., 2014) and lesion experiments (Hunsaker et al., 2013) that consider the differential function of the MEC and LEC, the full transverse axis of CA1 can thus be seen as an organizational framework generating a combined representation of the overall context (environment and location within it) and content of experience.

## Synthesis

Reviewing Editor: Liset Menendez de la Prida, Instituto Cajal CSIC Decisions are customarily a result of the Reviewing Editor and the peer reviewers coming together and discussing their recommendations until a consensus is reached. When revisions are invited, a fact-based synthesis statement explaining their decision and outlining what is needed to prepare a revision will be listed below. The following reviewer(s) agreed to reveal their identity: Stefan Remy, Menno Witter

Your ms was evaluated by two independent reviewers and myself. We all agree there is value in your data that merit publication. However, we feel you may need to further clarify some specific points:

- First, you may need to further clarify on numerical analysis and interpretation of the preferential projections of CA3 and entorhinal cortex to proximal CA1. This point is largely discussed by reviewer #2. In particular you need to address issues related to the proximo-distal CA3 to CA1 projection and stellate(reelin+) versus pyramidal(CB+) layer II cells from entorhinal cortex. You may need to discuss whether CA2 is confunding interpretation and consider the fact that layer II stellate cells may be acting over SLM-interneurons.

- A second methodological point concerns different tropism and pathway bias of rabies virus, as well as appropiate definition of starter cells/regions. Both reviewers raised concerns regarding these points that you should carefully address.

- Third, we all agree that VSD data are not convincing due to spatial and temporal resolution. You need providing more convinging data, or we may advocate removing this part. I understand such a data may be relevant so I challenge you to address the issues raised.

I copy below the reports by both reviewers. You should address carefuly the three points I synthetize above but also go over reviewers' report and clarify.

R1's comments:

My main concern is that the method -monotransynaptic tracing - may by itself lead to a bias towards specific inputs. As far as I know convincing data supporting an activity dependence of trans-synaptic labeling was largely absent until recently (Beier et al., 2017), although this issue has been heavily discussed in the field. The authors should add a brief section in which they discuss potential bias resulting from different in-vivo firing rates of different neuron types. Recordings from subicular neurons for example suggest higher mean firing rates during behavior than is observed in other putative input regions (Sharp and Green 1994, O'Mara et al 2000).

In addition there is the issue of neurotropism. For example it has been found that primary sensory neurons might not be effectively infected by RV. Recent data obtained in somatosensory cortex (Albisetti et al., 2017) further supports the view that some neuron types show resistance to rabies infection.

Both issues could in principle lead to a bias in the comparative assessment of input contributions. It would be good if the authors mention this more clearly in the discussion section.

My last point concerns the relatively low sampling rate of the VSD recordings (<200 Hz) and intrasubicular recurrent excitatory connections. Although only few neurons should be activated by the uncaging of MNI glutamate, it is possible that intrasubicular recurrent excitatory connections lead to a buildup of activity that is required for effective propagation of the signal into CA1 (which can not be temporally resolved). Could different excitation strength in CA1 with different distance from the CA1 border could be explained by different local intrasubicular excitatory connectivity (Böhm et al., 2015)?

R2's comments:

The first issue that I would like the authors to be more clear about relates to the numerical analysis and interpretation of the preferential projections of CA3 to proximal CA1. In the abstract and in the summary statement at the end of the introduction it is stated that there is a strong preference for ca3 inputs to proximal CA1, but in the results and in the significance statement I read that there is a preferred projection from distal CA3 to proximal CA1. The latter observation is not new, although it has not been incorporated in the generally accepted, what the authors refer to as the canonical wiring diagram. The first statemen that there is an overall preference of CA3 to proximal CA1 connections is much more interesting and should be elaborated on. Moreover, the apparent lack of projections from CA3c to CA1, seems to contradict previous findings from among others Ishizuka et al, cited by the authors, who report that this area projects to distal CA1. In this context, the authors might consider to discuss the recently reported particular role of the proxima CA3 to distal CA1 in non-spatial recognition memory (Nakamura NH, Flasbeck V, Maingret N, Kitsukawa T, Sauvage MM. Proximodistal segregation of nonspatial information in CA3: preferential recruitment of a proximal CA3-distal CA1 network in nonspatial recognition memory. J Neurosci. 2013 Jul 10;33(28):11506-14); Although the authors refer to this paper as a general reference with respect to transverse differences, they do not this potential issue that what seems a non-exiting or weak pathway has a relevant and specific role to play), for example in the text around line 365. Note that in the sentences around 345, the suggestion emerges that area CA3c does not project to CA1 at all. This is corrected in the subsequent more detailed description, but still, the reader who is not that knowledgeable with hippocampal circuitry might get the wrong impression.

The quantitative data apparently support the presented conclusion, however, there is a lack of data excluding that the proximal CA1 injections did not include parts of CA2, which might have resulted in a strong overestimation of CA3 inputs to proximal CA1.

The quantification as described, depends strongly on the ability to define the number of starter cells. This is not trivial, and this influences the data strongly. Although the authors in the method section describe this as a trivial issue, which we know it is not, they recognize this in the sentence starting in line 373: 'We did not quantify local excitatory connections in CA1 ipsilateral to the injection sites as labeled CA1 excitatory cells around the injection site could not be distinguished as primary versus secondarily infected by rabies in Camk2a-Cre; TVA cases. Adding the fairly well established differences in tropism for certain connections and celltypes, how sure are the authors that counting input cells based on this experimental approach is reflecting reality? In addition, how much does the number of input neurons to a single neuron, or the input index tell us about the functional effect of a connection?

Can the authors comment on the rather surprising and if true, potentially important finding that MEC inputs to CA1 are 35% larger than those from LEC. Can this be biased by position of injection sites or again tropism differences?

Following up on this, the authors aim to provide supportive electrophysiological support for their tracing-based claim on proximal subicular return projections to distal CA1. They claim that the optic/VSD results strongly support their findings of a gradient of projections, strong in distal AC1 weaker in proximal CA1; however, all they show is that prox sub stim results in a very tiny signal in a voxel located deep to the pyramidal layer of distal CA1. A few voxels more proximal of there, no signal is apparent in any of their datasets. The selected voxel is so close to the CA1/Sub border that one could very well be looking at local sub connectivity, similar to what is seen in case of more distal Sub stim, since at the border region between CA1 and Sub, there are many subicular neurons deep to the pyramidal cell layer of CA1. In addition, being so close to the border, how can they exclude to stimulate one or several of the dispersed CA1 neurons overlying the most proximal part of Sub? These data are unfortunately not convincing and I strongly suggest to generate more convincing data or to remove them from the paper. In case the authors feel strongly about keeping the VSD data in, provide quantification of the latencies between stim and local Sub versus presumed CA1 responses (line 484). Looking at the extended figure, I do see some interesting patterns that might support the claims made by the authors, so why did you chose to have this figure as extended material over Fig 7?

On page 19, top, the authors report that in several of their cases they find neurons in layer II of EC, both in MEC and in LEC that project to CA1. They claim that in both EC subdivisions these are stellate cells, based on morphology. Looking at figure 3, I find that insufficiently documented. It has recently been claimed that layer II CB+ neurons project to CA1 (Kitamura et al 2014, Science 343, 896). However, stellate cells are considered to be reeling neurons, not projecting to CA1. So, the authors should rephrase their conclusion, being more careful, or they should provide immune-labeling for Reelin and CB. By the way, it is now well accepted that the main class of layer II LEC reelin positive neurons are not stellate cells but fan cells

It is unclear to me what the authors mean/are implying with the sentence starting in line 443: 'Thus, SUB sends a major projection to CA1 neurons in a highly topographic manner. The sharpness of the gradient yields a SUB to distal CA1 connectivity strength that surpasses that of

the direct connections from LEC to distal CA1.' Moreover, how was this assessed/quantified?

The extended figures, in particular figure 1-1 is of relevance in view of the issue of what are starter cells; in case of distal CA1 tracing, can the authors convincingly show that no deep subicular neurons are part of the starter cell population? I am not convinced so would ask for higher resolution figures including clear criteria how to delineate CA1 from sub.

The third main conclusion derived from the data, starting on line 523, in particular the second part, ‘have approximately equal sensitivity to both total MEC/LEC input and CA3 input’. is not supported/substantiated by experimental data, so should be rephrased.

I strongly suggest the authors to rephrase the last sentence of the paper. What do they mean by this: that there is some kind on integrative representation in CA1, i.e. what is meant with combined? Be more specific where such combined representation is to be expected, at the neuronal level, at the network level, if so does CA1 have the necessary intrinsinc connectivity matrix to connect proximal to distal?

Minor issues to consider

Line 89, replace inner with middle molecular layer; the inner is the target of crossed and associational hilar projections.

Line 92, Layer III does not project to CA3 but does project to proximal and distal subiculum.

Line 95/96, it would be fair to change this sentence since this pathway has been reported already several years before (Seress L, Abrahám H, Lin H, Totterdell S. Nitric oxide-containing pyramidal neurons of the subiculum innervate the CA1 area. Exp Brain Res. 2002 Nov;147(1):38-44.)

Line 157 and following: I am not sure that the argument for survival being appropriate is supported by observing weak inputs being labeled, since weak or not is based on previous data, and the current data are used to argue, at least in part against the traditional concept/interpretation of weak or not weak inputs. One example of a potential problem is indicated in the paper, where in line 324 inputs to CA1 from the reuniens are listed as weak, whereas from published data it is obvious that synaptic density of entorhinal and reuniens inputs to AC1 might be of equal density, although this has never been thoroughly quantified. In relation to this, why report data on the sparse inputs to CA1 form MS and raphe andnot from reuniens; are th elater actually not as sparse as proposed.

The paper would improve is all detailed descriptions of methods in the results and figure legends would be removed. One of multiple examples are the lines 313 - 318.

Line 327, what are postsynaptic starter cells; how are they different from starter cells.

Line 367 is the first one where it is made explicit that all experiments address dorsal hippocampus. In view of the strong emphasis on dorsoventral functional differences, in addition to the transverse ones addressed in this paper, it might be good to emphasize this earlier in the paper, even in the abstract.

Line 457, ‘but’ should be ‘and’

Line 508, Witter 2007 does not report that distal CA3 is more heavily EC innervated than proximal parts at least not in the context of what the authors mean. It was reported that the part of CA3c, due to its physical location within C-shaped DG, does not receive much EC inputs.

Line 738 correct typo: references

In the reference list Xu et al is mentioned twice as two different ref a and b. This is obviously the same reference and their reference database is playing tricks (they have two entries of an incomplete and a complete version.). Please correct
