# Supplementary figures and images for: Opposing and Complementary Topographic Connectivity Gradients Revealed by Quantitative Analysis of Canonical and Noncanonical Hippocampal CA1 Inputs
Source: eNeuro. 2018 Jan 30;5(1):ENEURO.0322-17.2018. doi: 10.1523/ENEURO.0322-17.2018 (PMC5790753; doi:10.1523/ENEURO.0322-17.2018)

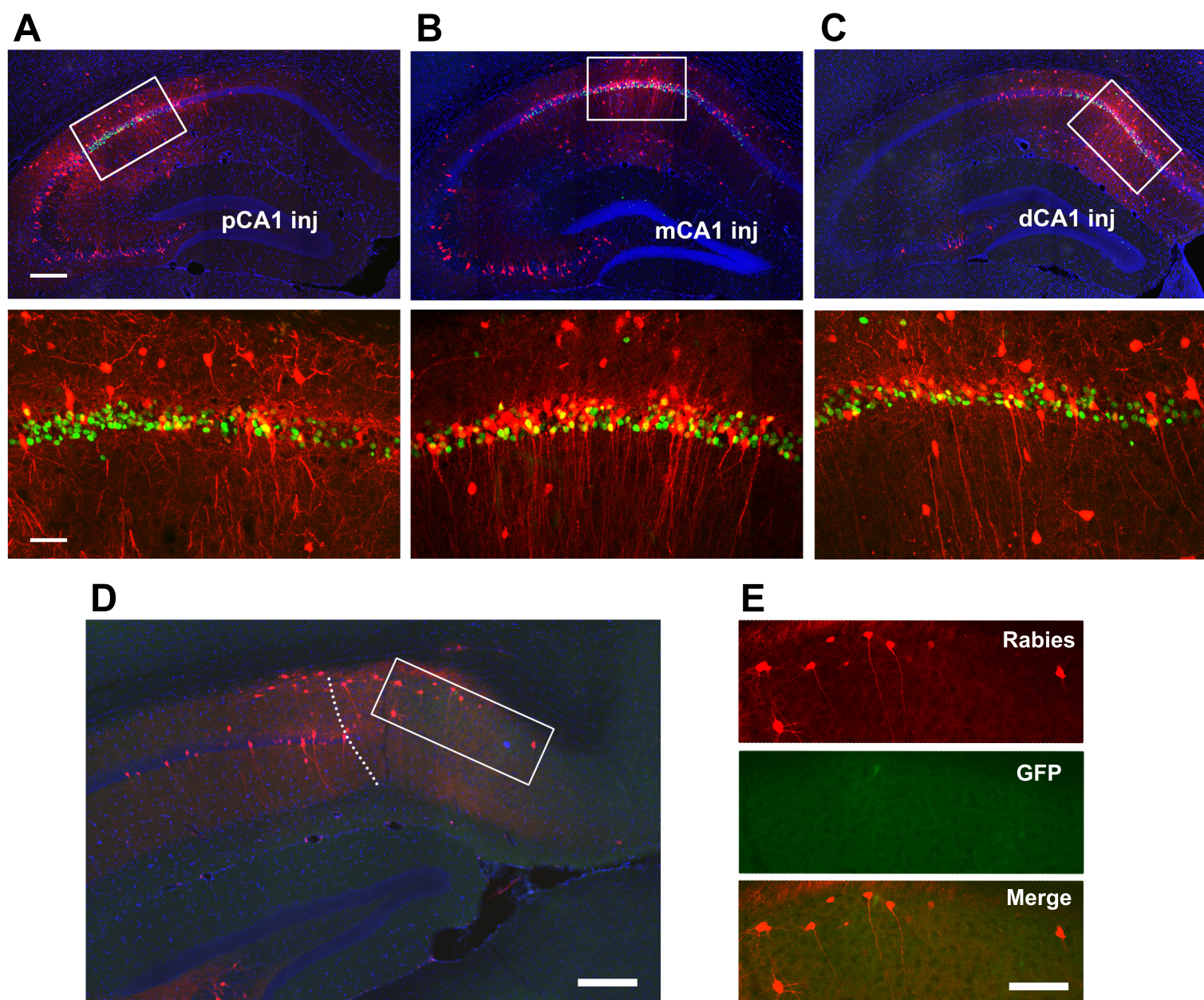

Figure 1-1

Supplement: Extended Data Figure 1-1 — Viral labeled neurons in CA1 injection sites. A–C, Example images show the viral pressure injection sites in proximal CA1 (pCA1), intermediate CA1 (mCA1), and distal CA1 (dCA1), respectively. The scale bar (200 μm) applies to the top panels of A, B, and C. Bottom panels are enlarged views of the white box region in their corresponding panels on top. Histone-tagged GFP expression of AAV helper virus is green, and mCherry expression of rabies virus is red. Double-labeled starter neurons appear yellow. The scale bar (50 μm) applies to the bottom panels of A, B, and C. D, A subiculum slice that is closest to the injection site in dCA1. The dotted line delineates the CA1 and subiculum border, which is defined by the abrupt change of pyramidal cell layer density. Scale bar = 200 μm. E, Enlarged view of the white box in D; rabies-labeled subiculum neurons are shown in red; there is no GFP expression (shown in green) of AAV helper virus in subiculum neurons. Scale bar = 100 μm. Download Figure 1-1, PDF file. [file sup_enu-eN-NWR-0322-17-s04.pdf]

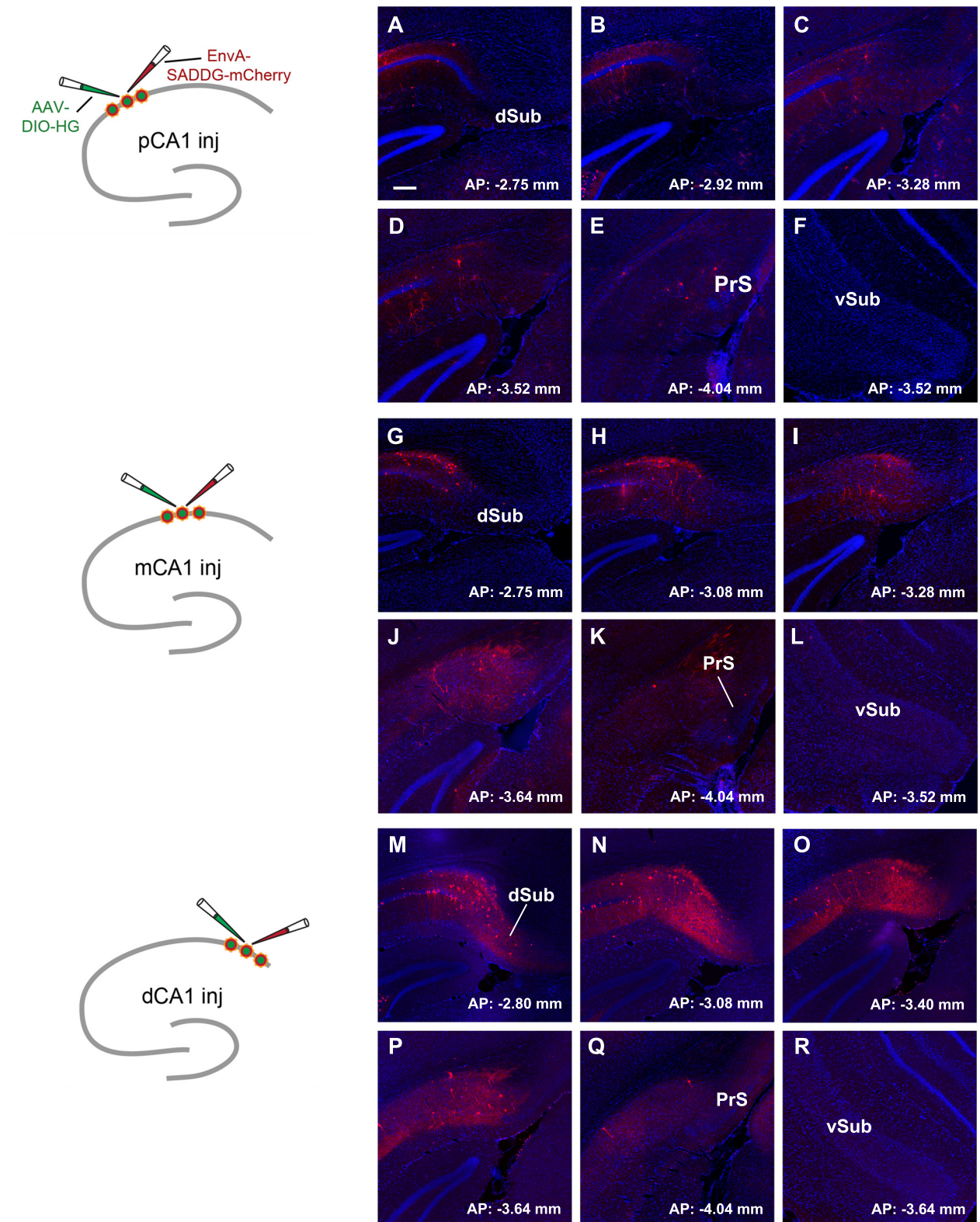

Figure 4-1

Supplement: Extended Data Figure 4-1 — Rabies tracing reveals noncanonical subiculum complex inputs to different CA1 subfields. A–D, Labeling of CA1-projecting subicular neurons in dorsal subiculum (dSub) in different sections along the rostral-caudal axis. The scale bar (200 μm) in C applies to all other panels. E, Rabies-labeled neurons in the presubiculum (PrS) and parasubiculum indicate that these neurons directly project to proximal CA1. F, No labeled neurons in ventral subiculum (vSub) indicate no direct input from vSub to distal CA1. G–L and M–R are formatted similarly to A–F, for rabies tracing from intermediate and distal CA1. Download Figure 4-1, PDF file. [file sup_enu-eN-NWR-0322-17-s03.pdf]
