# Supplementary material for: Opposing and Complementary Topographic Connectivity Gradients Revealed by Quantitative Analysis of Canonical and Noncanonical Hippocampal CA1 Inputs
Source: eNeuro. 2018 Jan 30;5(1):ENEURO.0322-17.2018. doi: 10.1523/ENEURO.0322-17.2018 (PMC5790753; doi:10.1523/ENEURO.0322-17.2018)
Supplement: Extended Table 1-1 — Download Table 1-1, DOCX file. [file sup_enu-eN-NWR-0322-17-s01.docx]

**Table 1-1. Statistical comparisons of input strengths to pCA1, mCA1, and dCA1**

| 1 | **Figure 6A comparisons** | Statistic Method | Significance | P value |
| --- | --- | --- | --- | --- |
|  | **Compare CA3a, CA3b, and CA3c inputs to pCA1** | One-way ANOVA | ** | P=0.0058 |
|  | CA3a vs CA3b | Post-hoc Tukey | ns |  |
|  | CA3a vs CA3c | Post-hoc Tukey | ** | P<0.01 |
|  | CA3b vs CA3c | Post-hoc Tukey | ns |  |
|  | **Compare CA3a, CA3b, and CA3c inputs to mCA1** | One-way ANOVA | * | P=0.0403 |
|  | CA3a vs CA3b | Post-hoc Tukey | ns |  |
|  | CA3a vs CA3c | Post-hoc Tukey | * | P<0.05 |
|  | CA3b vs CA3c | Post-hoc Tukey | ns |  |
|  | **Compare CA3a, CA3b, and CA3c inputs to dCA1** | One-way ANOVA | ** | P=0.0014 |
|  | CA3a vs CA3b | Post-hoc Tukey | ** |  |
|  | CA3a vs CA3c | Post-hoc Tukey | ns |  |
|  | CA3b vs CA3c | Post-hoc Tukey | ** |  |
|  | **Compare input from CA3a to pCA1, mCA1, and dCA1** | One-way ANOVA | *** | P=0.0005 |
|  | pCA1 vs mCA1 | Post-hoc Tukey | ns |  |
|  | pCA1 vs dCA1 | Post-hoc Tukey | *** | P<0.001 |
|  | imCA1 vs dCA1 | Post-hoc Tukey | * |  |
|  | **Compare input from CA3b to pCA1, mCA1, and dCA1** | One-way ANOVA | ** | P=0.0027 |
|  | pCA1 vs imCA1 | Post-hoc Tukey | ns |  |
|  | pCA1 vs dCA1 | Post-hoc Tukey | ** |  |
|  | imCA1 vs dCA1 | Post-hoc Tukey | ns |  |
|  | **Compare input from CA3c to pCA1, mCA1, and dCA1** | One-way ANOVA | ** | P=0.0019 |
|  | pCA1 vs mCA1 | Post-hoc Tukey | * |  |
|  | pCA1 vs dCA1 | Post-hoc Tukey | ** |  |
|  | mCA1 vs dCA1 | Post-hoc Tukey | ns |  |
| 2 | **Figure 6B comparisons** |  |  |  |
|  | **Compare contralateral CA3a, CA3b, and CA3c inputs to pCA1** | One-way ANOVA | *** | P=0.0002 |
|  | CA3a vs CA3b | Post-hoc Tukey | * |  |
|  | CA3a vs CA3c | Post-hoc Tukey | *** |  |
|  | CA3b vs CA3c | Post-hoc Tukey | * |  |
|  | **Compare contralateral CA3a, CA3b, and CA3c inputs to mCA1** | One-way ANOVA | ** | P=0.0033 |
|  | CA3a vs CA3b | Post-hoc Tukey | ns |  |
|  | CA3a vs CA3c | Post-hoc Tukey | ** |  |
|  | CA3b vs CA3c | Post-hoc Tukey | * |  |
|  | **Compare contralateral CA3a, CA3b, and CA3c inputs to dCA1** | One-way ANOVA | ns | P=0.1193 |
|  | **Compare input from contralateral CA3a to pCA1, mCA1, and dCA1** | One-way ANOVA | *** | P<0.0001 |
|  | pCA1 vs imCA1 | Post-hoc Tukey | * |  |
|  | pCA1 vs dCA1 | Post-hoc Tukey | *** |  |
|  | imCA1 vs dCA1 | Post-hoc Tukey | ** |  |
|  | **Compare input from contralateral CA3b to pCA1, mCA1, and dCA1** | One-way ANOVA | ns | P=0.1486 |
|  | **Compare input from contralateral CA3c to pCA1, mCA1, and dCA1** | One-way ANOVA | ns | P=0.2817 |
| 3 | **Figure 6C comparisons** |  |  |  |
|  | **Compare MEC and LEC inputs to pCA1** | t-test | ** | P=0.0026 |
|  | **Compare MEC and LEC inputs to mCA1** | t-test | * | P=0.0354 |
|  | **Compare MEC and LEC inputs to dCA1** | t-test | ** | P=0.0019 |
|  | **Compare MEC inputs to pCA1, mCA1, and dCA1** | One-way ANOVA | *** | P=0.0008 |
|  | pCA1 vs mCA1 | Post-hoc Tukey | * |  |
|  | pCA1 vs dCA1 | Post-hoc Tukey | *** |  |
|  | mCA1 vs dCA1 | Post-hoc Tukey | ns |  |
|  | **Compare LEC inputs to pCA1, mCA1, and dCA1** | One-way ANOVA | ** | P=0.0021 |
|  | pCA1 vs mCA1 | Post-hoc Tukey | ns |  |
|  | pCA1 vs dCA1 | Post-hoc Tukey | ** |  |
|  | mCA1 vs dCA1 | Post-hoc Tukey | * |  |
| 4 | **Figure 6D comparisons** |  |  |  |
|  | **Compare Subiculum inputs to pCA1, mCA1, and dCA1** | One-way ANOVA | *** | P=0.0009 |
|  | pCA1 vs mCA1 | Post-hoc Tukey | ns |  |
|  | pCA1 vs dCA1 | Post-hoc Tukey | *** |  |
|  | mCA1 vs dCA1 | Post-hoc Tukey | ** |  |
|  | **Compare Pre-subiculum inputs to pCA1, mCA1, and dCA1** | One-way ANOVA | * | P=0.0301 |
|  | pCA1 vs mCA1 | Post-hoc Tukey | ns |  |
|  | pCA1 vs dCA1 | Post-hoc Tukey | * |  |
|  | mCA1 vs dCA1 | Post-hoc Tukey | ns |  |
| **5** | **Data comparison of contralateral CA1 inputs** |  |  |  |
|  | **Compare inputs from contralateral pCA1, mCA1, and dCA1 to pCA1** | One-way ANOVA | * | P=0.0400 |
|  | Contra pCA1 vs Contra mCA1 | Post-hoc Tukey | ns |  |
|  | Contra pCA1 vs Contra dCA1 | Post-hoc Tukey | ns |  |
|  | Contra mCA1 vs Contra dCA1 | Post-hoc Tukey | ns |  |
|  | **Compare inputs from contralateral pCA1, mCA1, and dCA1 to mCA1** | One-way ANOVA | ns | P=0.0752 |
|  | **Compare inputs from contralateral pCA1, mCA1, and dCA1 to dCA1** | One-way ANOVA | *** | P=0.0003 |
|  | Contra pCA1 vs Contra mCA1 | Post-hoc Tukey | ns |  |
|  | Contra pCA1 vs Contra dCA1 | Post-hoc Tukey | *** |  |
|  | Contra mCA1 vs Contra dCA1 | Post-hoc Tukey | ** |  |
|  | **Compare contralateral dCA1 inputs to pCA1, mCA1, and dCA1** | One-way ANOVA | ** | P=0.0100 |
|  | Contra pCA1 vs Contra mCA1 | Post-hoc Tukey | ns |  |
|  | Contra pCA1 vs Contra dCA1 | Post-hoc Tukey | ** |  |
|  | Contra mCA1 vs Contra dCA1 | Post-hoc Tukey | ns |  |
| 6 | **Data comparison of median raphe inputs** |  |  |  |
|  | **Compare median raphe inputs to pCA1, mCA1, and dCA1** | One-way ANOVA | *** | P<0.0001 |
|  | pCA1 vs mCA1 | Post-hoc Tukey | *** |  |
|  | pCA1 vs dCA1 | Post-hoc Tukey | *** |  |
|  | mCA1 vs dCA1 | Post-hoc Tukey | ns |  |
| 7 | **Data comparison of MS-DB inputs** |  |  |  |
|  | **Compare MS-DB inputs to pCA1, mCA1, and dCA1** | One-way ANOVA | * | P=0.0179 |
|  | pCA1 vs mCA1 | Post-hoc Tukey | * |  |
|  | pCA1 vs dCA1 | Post-hoc Tukey | ns |  |
|  | mCA1 vs dCA1 | Post-hoc Tukey | ns |  |
